# HIV-1 Tat Promotes Integrin-Mediated HIV Transmission to Dendritic Cells by Binding Env Spikes and Competes Neutralization by Anti-HIV Antibodies

**DOI:** 10.1371/journal.pone.0048781

**Published:** 2012-11-13

**Authors:** Paolo Monini, Aurelio Cafaro, Indresh K. Srivastava, Sonia Moretti, Victoria A. Sharma, Claudia Andreini, Chiara Chiozzini, Flavia Ferrantelli, Maria R. Pavone. Cossut, Antonella Tripiciano, Filomena Nappi, Olimpia Longo, Stefania Bellino, Orietta Picconi, Emanuele Fanales-Belasio, Alessandra Borsetti, Elena Toschi, Ilaria Schiavoni, Ilaria Bacigalupo, Elaine Kan, Leonardo Sernicola, Maria T. Maggiorella, Katy Montin, Marco Porcu, Patrizia Leone, Pasqualina Leone, Barbara Collacchi, Clelia Palladino, Barbara Ridolfi, Mario Falchi, Iole Macchia, Jeffrey B. Ulmer, Stefano Buttò, Cecilia Sgadari, Mauro Magnani, Maurizio P. M. Federico, Fausto Titti, Lucia Banci, Franco Dallocchio, Rino Rappuoli, Fabrizio Ensoli, Susan W. Barnett, Enrico Garaci, Barbara Ensoli

**Affiliations:** 1 National AIDS Center, Istituto Superiore di Sanità, Rome, Italy; 2 Novartis Vaccines & Diagnostics, Inc., Cambridge, Massachusetts, United States of America; 3 CERM, University of Florence, Florence, Italy; 4 Department of Biochemistry, University of Ferrara, Ferrara, Italy; 5 San Gallicano Hospital, Rome, Italy; 6 Novartis Vaccines & Diagnostics, Inc., Siena, Italy; 7 Department of Biomolecular Science, University of Urbino, Urbino, Italy; Institut Pasteur Korea, Republic of Korea

## Abstract

Use of Env in HIV vaccine development has been disappointing. Here we show that, in the presence of a biologically active Tat subunit vaccine, a trimeric Env protein prevents in monkeys virus spread from the portal of entry to regional lymph nodes. This appears to be due to specific interactions between Tat and Env spikes that form a novel virus entry complex favoring R5 or X4 virus entry and productive infection of dendritic cells (DCs) via an integrin-mediated pathway. These Tat effects do not require Tat-transactivation activity and are blocked by anti-integrin antibodies (Abs). Productive DC infection promoted by Tat is associated with a highly efficient virus transmission to T cells. In the Tat/Env complex the cysteine-rich region of Tat engages the Env V3 loop, whereas the Tat RGD sequence remains free and directs the virus to integrins present on DCs. V2 loop deletion, which unshields the CCR5 binding region of Env, increases Tat/Env complex stability. Of note, binding of Tat to Env abolishes neutralization of Env entry or infection of DCs by anti-HIV sera lacking anti-Tat Abs, which are seldom present in natural infection. This is reversed, and neutralization further enhanced, by HIV sera containing anti-Tat Abs such as those from asymptomatic or Tat-vaccinated patients, or by sera from the Tat/Env vaccinated monkeys. Thus, both anti-Tat and anti-Env Abs are required for efficient HIV neutralization. These data suggest that the Tat/Env interaction increases HIV acquisition and spreading, as a mechanism evolved by the virus to escape anti-Env neutralizing Abs. This may explain the low effectiveness of Env-based vaccines, which are also unlikely to elicit Abs against new Env epitopes exposed by the Tat/Env interaction. As Tat also binds Envs from different clades, new vaccine strategies should exploit the Tat/Env interaction for both preventative and therapeutic interventions.

## Introduction

HIV-1 preventative vaccines based on the HIV Env antigen have either failed or shown marginal protection [1], [2], [3], indicating that Env is not sufficient to protect against infection and that innovative approaches are needed. Approaches employing the HIV regulatory Tat protein have been shown to contain virus replication, preventing disease onset and/or progression in monkey models [4]–[9] (http://www.avip-eu.org, http://www.hiv1tat-vaccines.info). The Tat-based vaccine has then been advanced to preventative and therapeutic phase I trials showing safety and immunogenicity [10]–[12] and has recently demonstrated promising efficacy in a phase II therapeutic trial in HAART-treated subjects [13].

A large body of evidence provides the rationale for inclusion of Tat in novel HIV/AIDS preventative vaccine candidates. The Tat protein is released during acute infection of T cells by a leaderless secretory pathway [14]–[16] and is present in tissues and sera of infected individuals as well as on HIV virus particles [17]–[21]. Extracellular Tat is mostly bound to heparan sulfate proteoglycans (HSPG) of the extracellular matrix (ECM) [16], [17], and engages the arginine-glycine-aspartic acid (RGD)-binding integrins αvβ3, α5β1 and αvβ5 on activated endothelial cells and co-stains with these integrins in tissues from infected individuals [17], [22]–[28]. These integrins are highly expressed by DCs and macrophages, which are known: I) to be a main target of HIV at the portal of entry, II) to favor the establishment and spreading of the infection, and III) to become viral reservoirs in chronic infection. Thus, these cells appear to be key to initiate a self-propagating infection at the portals of entry, which, in turn, is essential to establish a self-renewing virus reservoir in regional lymph nodes required for a persistent infection. By targeting DCs, extracellular Tat also induces their maturation with cytokine production and proteasome activation [29]–[31], leading to increase and hierarchy modification of CTL epitope presentation of heterologous antigens including HIV [31], [32]. This may result in excessive and improper immune stimulation that prepares target cells for virus propagation. Finally, extracellular adherent Tat can efficiently increase virus transmission to T cells [33].

Of note, anti-Tat Abs, which are uncommon in natural infection and present in about 20% of asymptomatic individuals, were reported to correlate with non progression to AIDS in humans and shown to inhibit HIV infection in vitro [34]–[38]. Further, promising efficacy data were obtained in non-human primates, where prevention or containment of infection at the portal of entry or reduction of peak viral load and CD4 decay were observed in monkeys immunized parenterally with Tat combined with Env and challenged with SHIV89.6P or SHIVSF162 [39]–[41].

Therefore, novel vaccine strategies combining Tat and Env antigens are being developed with the aim of inducing broad cellular and humoral immune responses able to kill early the infected cells, as well as to neutralize infectious virions, acting together to dampen or to block initial HIV infection and dissemination (http://www.avip-eu.org) [42].

Here we show that mucosal (nasal) vaccination with Tat and trimeric V2-deleted (ΔV2) Env proteins combined prevents virus spreading from the portal of entry to regional lymph nodes in monkeys and, consequently, virus dissemination and establishment of a virus reservoir. This is because Tat forms with Env a new virus entry complex by binding the V3 loop of Env and by using RGD-binding integrin-receptors to target and to promote virus entry and productive infection of DCs and likely other cell types at the portals of entry expressing RGD-binding integrins. This occurs with both R5 and X4 HIV variants and with trimeric Env from different clades. Of note, by binding to Tat, Env appears to be shielded against HIV neutralizing Abs, which in the presence of Tat are much less effective. In contrast, in the presence of Tat, Abs against both Tat and Env restore and further increase neutralization of HIV entry into DCs and cell infection. This is observed with both natural and vaccine-induced anti-Tat Abs from sera of HIV-infected individuals and with sera from Tat/Env vaccinated monkeys.

These data indicate that both anti-Tat and anti-Env Abs are required to block HIV acquisition and spreading and have key implications for new strategies in both preventative and therapeutic HIV vaccine development.

**Figure 1:**
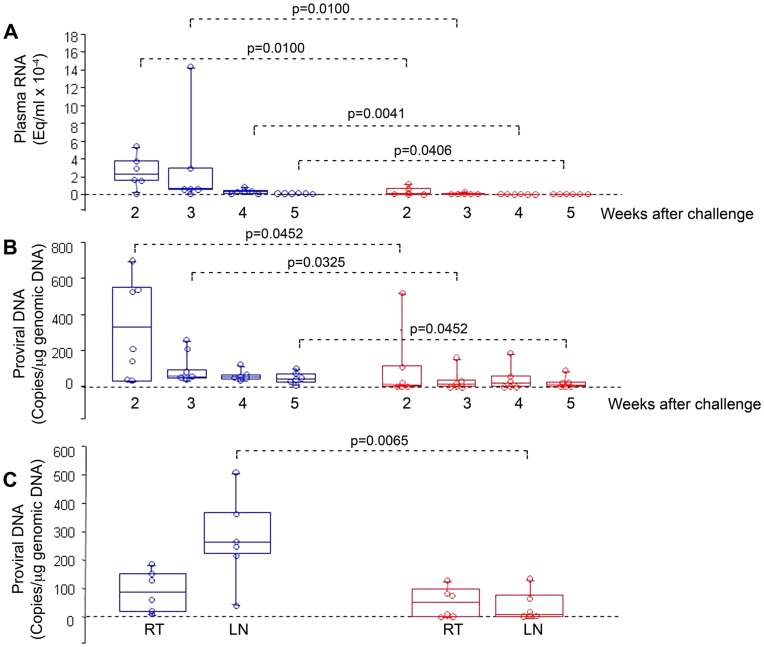
Virological outcome in Tat/Env-vaccinated or control monkeys after intrarectal challenge with the SHIV_SF162P4cy_ (70 MID_50_). Box plots of (A) Viral RNA, (B) proviral DNA in blood at 2, 3, 4 and 5 weeks after challenge, respectively; and (C) proviral DNA at week 4 after challenge in rectal tissue (RT) and inguinal lymph nodes (LN). Statistical analysis was performed by the one-sided Wilcoxon rank sum test. Red: monkeys vaccinated with Tat/Env (n = 6); blue: control animals (n = 6).

## Materials and Methods

### Ethics Statement

Adult male cynomolgus monkeys (*Macaca fascicularis*) were housed at the National AIDS Center, Istituto Superiore di Sanità (ISS), according to the European guidelines for non-human primate care (ECC, Directive No. 86–609, Nov. 24, 1986). Animal experiments were approved by the Quality and Safety Committee for Animal Trials of the ISS. All clinical procedures were performed upon anesthesia with 7–10 mg/kg Zoletil (a combination of Tiletamine and Zolazepam).

Studies in humans were conducted according to the principles of the Declaration of Helsinki and all subjects signed an informed consent. The ISS T-002 clinical trial (ClinicalTrials.gov NCT00751595) [13] was approved by the competent authority (General Director of the Coordinator Clinical Center, Policlinico of Modena, Modena) and by the Ethics Committees of each participating clinical center (Policlinico of Modena, Modena; Arcispedale S. Anna, Ferrara; Istituti Fiosterapici Ospitalieri San Gallicano, Rome; Policlinico of Bari, Bari; Ospedale S.M. Goretti Latina; Fondazione S. Raffaele, Milan; Ospedale S. Maria Annunziata, Florence; Ospedale Luigi Sacco, Milan; Spedali Civili, Brescia; Ospedale A. di Savoia, Turin).

**Figure 2:**
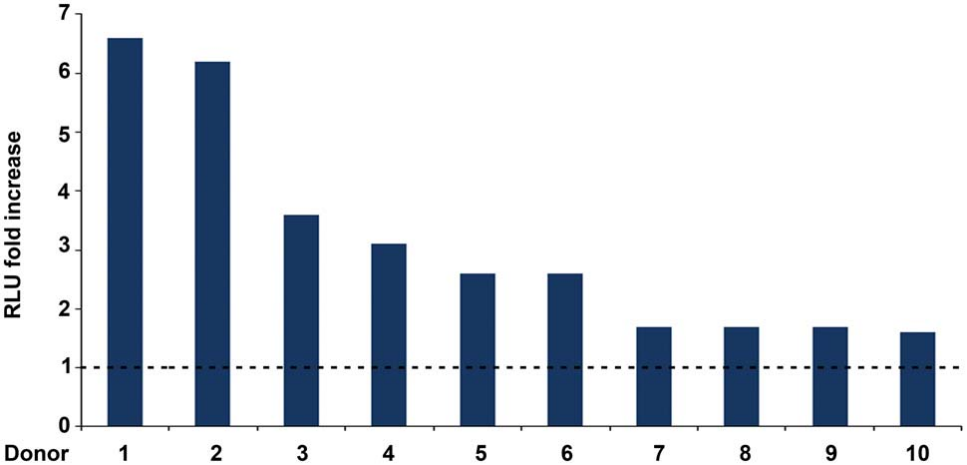
Enhancement by soluble Tat of HIV-1 infection in MDDCs. Peak infection fold increase reached 3–8 days after infection with the R5 pSF162LUC in MDDCs from 10 different donors. The virus was pre-incubated with 1 µM cys_22_ Tat or with PBS-0.1% BSA (control buffer). Fold increase were calculated as the ratio between the RLU from MDDCs infected with virus pre-incubated with cys_22_ Tat and RLU from MDDCs infected with virus pre-incubated with control buffer (baseline RLU: 298.95) (both values had been previously subtracted of the uninfected MDDCs RLU background).

**Figure 3:**
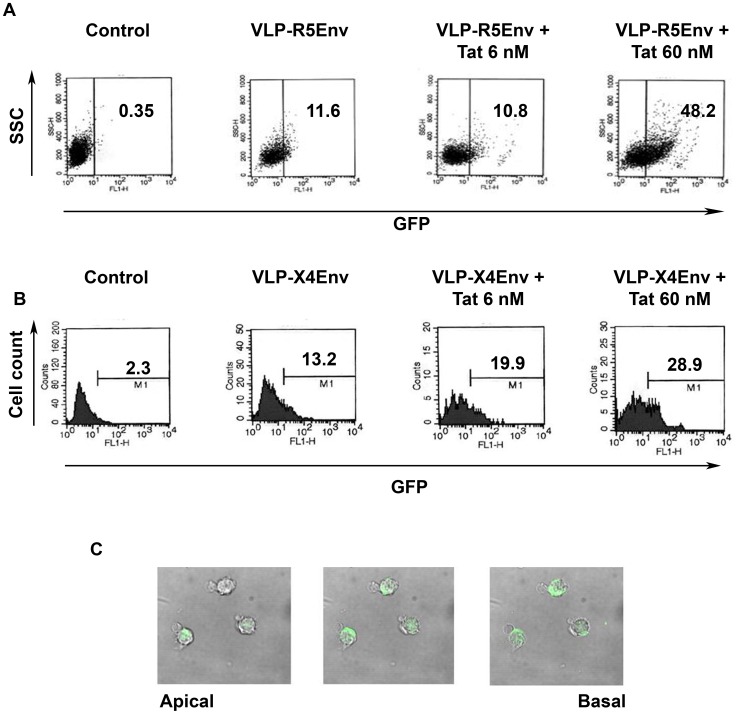
Tat-mediated entry of VLPs expressing R5 or X4 Env into MDDCs. (**A**) and (**B**): entry in immature MDDCs of null-VLPs (control) or (A) VLPs expressing R5-Env (HIV**-**1 BaL) (VLP-R5Env) or (B) VLPs expressing X4-Env (HIV**-**1 HXBc2) (VLP-X4Env), in the absence or presence of increasing concentrations of cys_22_ Tat after 4 h of incubation, evaluated by flow cytometry. Results are shown as either dot-plot (A) or histograms (B). SSC: side scatter. Numbers in the insets represent the percentage of GFP positive cells. (**C**) Confocal microscopy analysis of VLP-R5Env entry into MDDCs in the presence of cys_22_ Tat (60 nM) after 4 h of incubation (0.2 µm optical sections form the apical to the basal cell side).

### Production and Purification of Recombinant Biologically Active HIV-1 Tat, cys_22_ Tat and Env Proteins

The HIV-1 Tat and cys_22_ Tat proteins from human T lymphotropic virus type IIIB-BH-10 (subtype B) were expressed in *Escherichia coli,* purified to homogeneity by heparin affinity chromatography and reverse HPLC, and handled as described previously [15], [16], [29]. In brief, to prevent oxidation that occurs easily because Tat contains seven cysteines, the Tat protein was stored lyophilized at -80°C and resuspended in degassed buffer before use. To prevent attachment of the protein to surfaces, plastic tips and vials were previously rinsed in 0.1% PBS-BSA (Tat buffer) or in RPMI 1640 supplemented with 20 mM HEPES (Sigma-Aldrich, St. Louis, MO), 100 U/mL penicillin, 100 µg/mL streptomycin, 2 mM L-glutamine (Life Technologies, Paisley, U.K.), and 15% FBS (HyClone Laboratories, Logan, UT) (complete medium). In addition, because Tat is also photo- and thermo-sensitive [15], [16], [29], the handling of the protein was always performed in the dark and on ice. Different protein lots were used with reproducible results. In all lots endotoxin concentration was below the detection limit (<0.02 EU/µg), as determined by the Lymulus Amoebocyte Lysate analysis (Pyrochrome, Associates of Cape Cod, Falmouth, MA). The purified Tat protein was fully biologically active, as determined by the rescue assay on HLM-1 cell line carrying a Tat-defective HIV provirus [15], [16], by the induction of transcription in TZM-bl cells, which contain a luciferase reporter gene under the transcriptional control of the HIV LTR and are commonly used to assess HIV infectivity (Fig. S1), as well as by Tat uptake by monocyte-derived dendritic cells (MDDCs) evaluated by intracellular staining for Tat in flow cytometry [29]. This assay constitutes the potency test for HIV-1 Tat protein, since it is highly specific for the reduced form of Tat (uptake does not occur with the oxidized form), and is strictly dose-dependent, allowing a precise determination of the content of active protein in the preparation. Cys_22_ Tat is a clade B Tat protein carrying a cysteine to glycine substitution at position 22 (cys_22_ Tat), which renders the protein transactivation-silent (Fig. S1) [43] although is taken up by MDDCs as well as wild type (wt) Tat [29].

**Figure 4:**
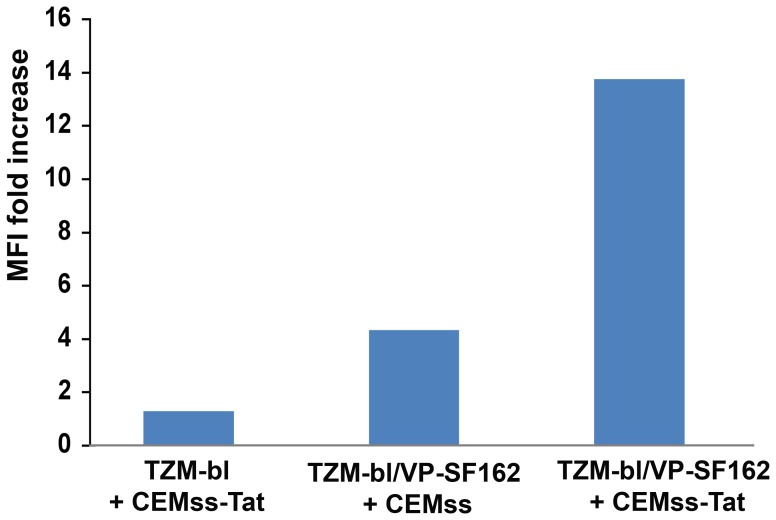
Tat released by producing T cells increases entry and infection of a Tat-independent replication-incompetent SF162 pseudovirus. CEMss cells were either infected (CEMss-Tat) or not infected (CEMss) with VSV-G/HIV. After 24 h cells were co-cultured for 4 days with TZM-bl cells in the presence (TZM-bl/VP-SF162) or absence (TZM-bl) of a Tat-independent GFP-expressing single-cycle HIV-1 SF162 virus (VP/SF162), and then GFP expression evaluated by flow cytometry. Results are expressed as MFI fold increase with respect to the TZM-bl + CEMss co-culture (baseline MFI: 1.9).

**Figure 5:**
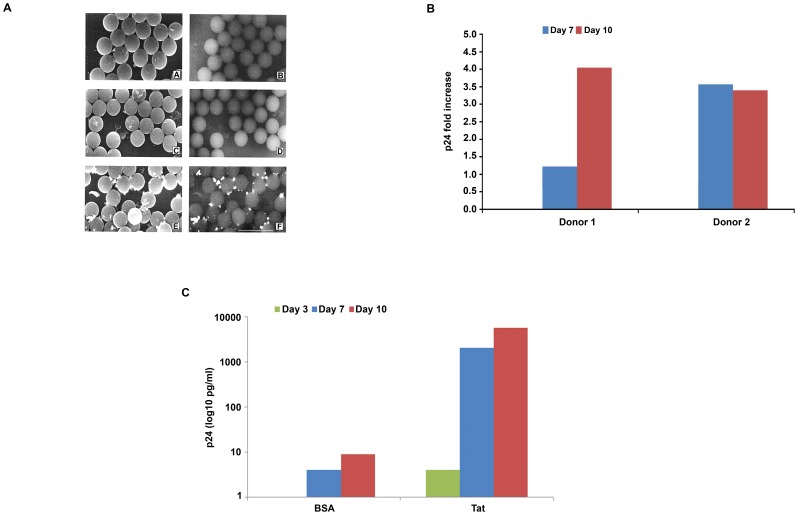
Adherent Tat binds HIV particles and increases MDDC productive infection and transmission to T cells. (**A**) Immuno-scanning electron microscopic analysis of HIV bound to Tat-coated latex beads. Images obtained with secondary electrons (left panels) or with backscattered electron (right panels) are shown. Latex beads were incubated with control buffer and HIV (*A, B*); with Tat and medium (*C, D*); or with Tat and HIV (*E, F*). Bar represents 5 µm. (**B**) MDDC infection with HIV SF162 on adherent wt Tat at different time points after infection. Infection of MDDCs from 2 different donors was carried out on plates coated with wt Tat (0.01 µM for donor 1, 0.1 µM for donor 2) or control buffer. Infection fold increase was calculated as the ratio between the content of p24 antigen detected in the supernatants of MDDCs seeded on adherent Tat and that detected in the supernatants of MDDCs seeded on control buffer (baseline, for donor 1, p24 was 154 and 119 pg/mL at day 7 and 10, respectively; for donor 2, p24 was 38 and 159 pg/mL at day 7 and 10, respectively). (**C**) Infection of PHA-activated CD4 T cell blasts with supernatants from MDDCs infected with HIV SF162 on plates coated with Tat wt (0.1 µM) or with control buffer (BSA). Infection was evaluated at the indicated time points by measuring p24 antigen in the culture supernatants in duplicate wells.

Monomeric and trimeric wt or ΔV2 Env molecules from clade B SF162 HIV have been described elsewhere [44]. For the ΔV3 Env molecule, the partial V3 loop deletion was generated by deleting residues 301 to 315 and 317 to 319, and by introducing an Ala to Gly and an Ile to Ala substitution at position 316 and 320, respectively, in SF162 gp140 [45].

### Monkey Studies

Adult healthy male cynomolgus monkeys (*Macaca fascicularis*) were imported from the Mauritius breeding colony and were matched for age and weight. All monkeys were negative for infections with simian immunodeficiency virus (SIV), type D simian 1, 2, 3, 5 retroviruses (SRV) and simian T cell leukaemia (STLV-I) viruses, simian herpes B virus, cytomegalovirus, Ebola and Marburg viruses. They were housed at the National AIDS Center, ISS, in an authorized P3 facility in single stainless steel cages according to the National (Ministry of Health D.L. 27/1/1992 n. 116) and European guidelines for non-human primate care (ECC, Directive No. 86–609, Nov. 24, 1986; Refinement, Reduction and Replacement towards the use of animals for scientific procedures. 99/167/EC: Council Decision of 25/1/99). The temperature was maintained at 21–23°C and humidity ranged from 50 to 60% with 10–15% air changes per h. The light cycle was 12-h light/12-h dark. Animals were fed a commercial maintenance chow (Rieper, Mucedola s.r.l., Settimo Milanese, Italy) supplemented biweekly with fresh fruit. Water was supplied *ad libitum*. Animal experiments were approved by the Quality and Safety Committee for Animal Trials of the ISS. All clinical procedures were performed upon anesthesia with 7–10 mg/kg Zoletil (a combination of Tiletamine and Zolazepam). Animals were examined clinically and weight and rectal temperature were measured while under ketamine hydrochloride anaesthesia (10 mg Kg^-1^, intramuscularly). Blood samples for haematological analysis and for immunological and virological assays were taken in the morning prior to feeding. All monkeys were observed daily for behavior, local reactions after immunizations, and clinical signs of disease. Serum biochemical/hematological parameters and animal weight were assessed on a regular basis, according to the blood drawing schedule. No local reactions were recorded after immunizations and all safety-related parameters evaluated were in the normal range at all times. All animals lived throughout the study.

**Figure 6:**
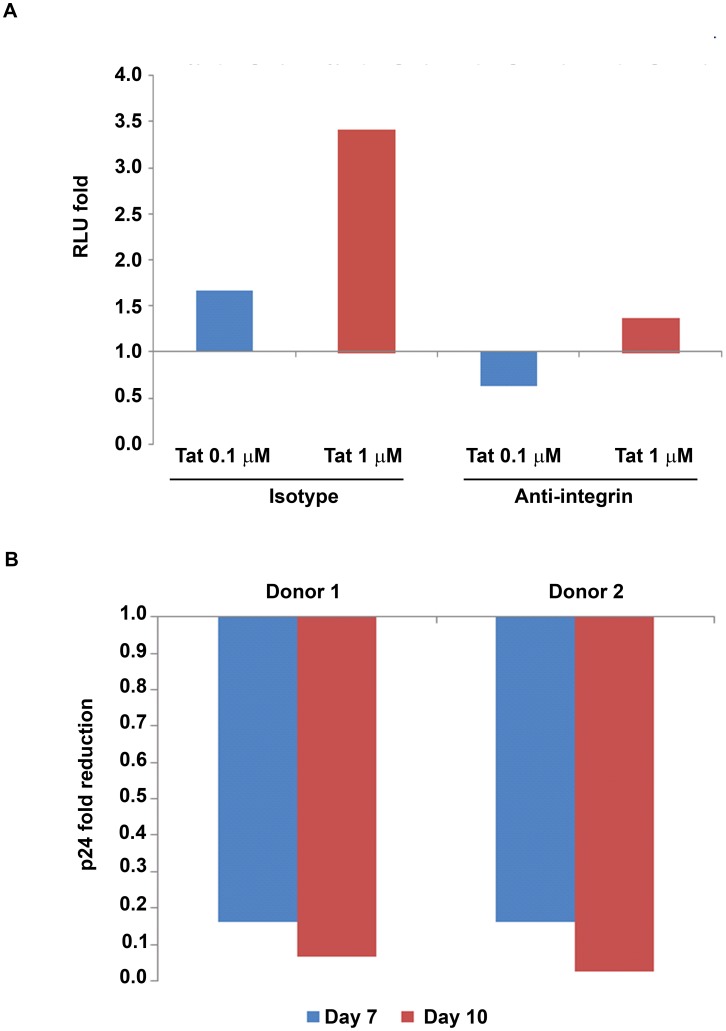
Anti-integrin antibodies block the enhancement of MDDC infection induced by soluble or adherent Tat. (**A**) pSF162LUC infection of MDDCs for 8 days in the presence of soluble cys_22_ Tat (0.1 or 1 µM) and a combination of mAbs directed against the α5β1, αvβ3 and αvβ5 integrins (10 µg/mL each) or the control isotype mAb (30 µg/mL). For each Tat concentration (Tat 0.1 µM, blu bar; Tat 1 µM, red bar) infection fold changes were calculated as the ratio between RLU from MDDCs pre-incubated with the control isotype mAb in the presence and in the absence of Tat (baseline RLU: 97) or as the ratio between RLU from MDDCs pre-incubated with anti-integrin mAbs in the presence and in the absence of Tat (baseline RLU: 112) (all values had been previously subtracted of the uninfected MDDC RLU background). Experiments were performed in duplicate. (**B**) Infection of MDDCs at different time points in the presence of coated wt Tat (0.01 µM for donor 1, 0.1 µM for donor 2) and of mAbs directed against the α5β1, αvβ3 and αvβ5 integrins (10 µg/mL each) or the control isotype mAb (30 µg/mL). Fold reduction of infection was calculated as the ratio between the content of p24 antigen detected in the supernatants of MDDCs seeded on adherent Tat in the presence of anti-integrin mAbs and that detected in the supernatants of MDDCs seeded on adherent Tat in the presence of the control isotype mAb (baseline, for donor 1, p24 was 186 and 479 pg/mL at day 7 and 10, respectively; for donor 2, p24 was 135 and 538 pg/mL at day 7 and 10, respectively). Experiments were performed in duplicate.

For immunization, biologically active HIV-1 Tat protein (10 µg) was mixed with trimeric ΔV2 Env protein (100 µg). The mixture was then given intranasally (150 µL per nostril) in the presence of the adjuvant LTK-63 (30 µg) [46] to 6 cynomolgus macaques (weeks 0, 4, 8), followed by subcutaneous injections (weeks 24 and 36) of Tat (10 µg) combined with trimeric ΔV2 Env (100 µg) proteins in the presence of the Alum adjuvant. Six additional monkeys received adjuvants only (controls). At week 44 all macaques were challenged intrarectally with 70 MID_50_ of the R5-tropic cynos-adapted SHIV_SF162P4cy_ (Table S1), which had been titrated intrarectally in 10 cynomolgus monkeys [41].

Inguinal lymph nodes and rectal mucosa samples were removed surgically under ketamine hydrochloride general anesthesia from all vaccinated and control monkeys at week 4 after the challenge. The tissues were therefore minced to obtain single cell suspension for detection of proviral DNA.

**Figure 7:**
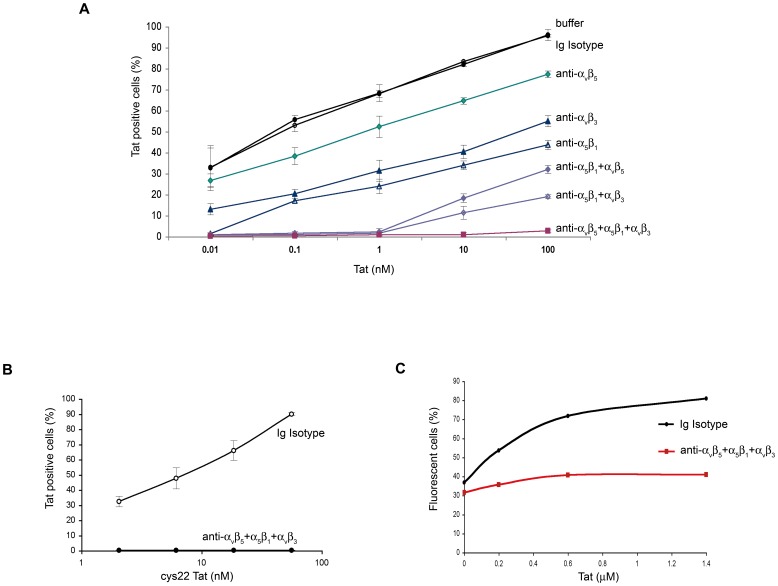
Entry of Tat or R5 Env-VLPs in MDDCs and block by anti-integrin antibodies. (**A**) Entry of wt Tat into MDDCs from 3–10 different donors (depending on the anti-integrin mAbs used), in the presence of mAbs against the indicated integrins alone or combined, a control isotype mAb, or nil (buffer). (**B**) Entry of cys_22_ Tat into MDDCs from 3 different donors and block by the combined anti-integrin mAbs versus an Ig control isotype mAb. The percentages of Tat positive cells +/− standard deviations are shown. (**C**) Entry of VLP-R5Env (BaL) in MDDCs in the presence of Tat and block by anti-integrin mAbs or an Ig control isotype mAb. The percentages of fluorescent cells are shown. A representative experiment out of 4 performed is shown.

SHIV RNA in plasma was quantitated by nucleic acid sequenced-based amplification using TaqMan Real-Time RT-PCR assay with a threshold limit for detection of 50 RNA Eq/mL as previously described [47]. DNA was extracted from whole blood using the QIAmp Blood Kit (QIAGEN, GmbH, Hilden, Germany) and from lymph nodes or rectal mucosa using the QIAmp DNA Mini Kit (QIAGEN, GmbH, Hilden, Germany). SHIV proviral copy number was determined using TaqMan Real-time PCR utilizing 1 µg of DNA and amplifying a region of 71 bp within the *gag* region of SHIV [48]. The lower limit of detection was 1 SHIV proviral copy/µg of DNA.

**Figure 8:**
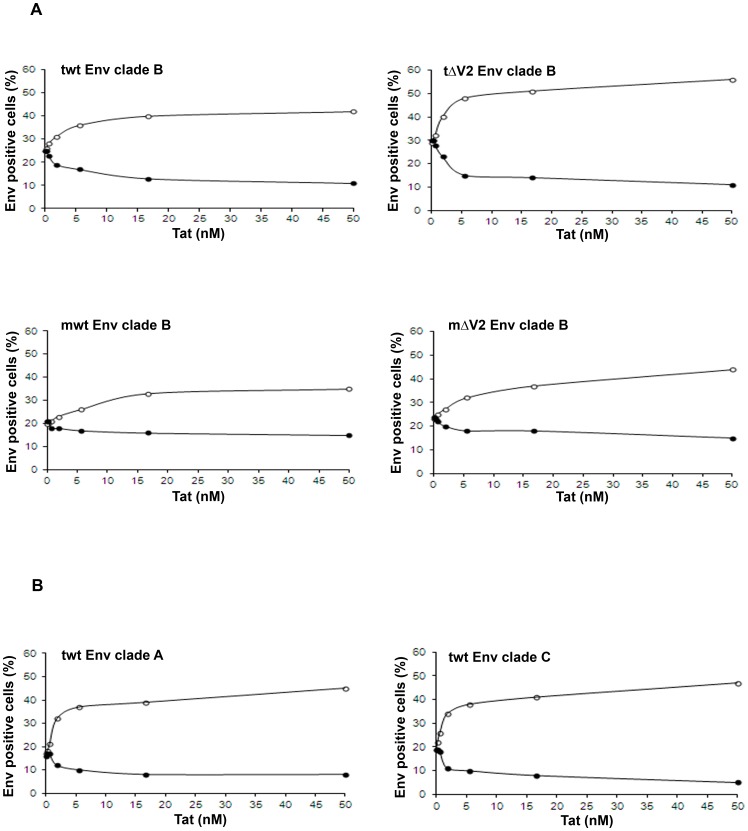
Tat-mediated entry of Env molecules from different clades in MDDCs and block by anti-integrins antibodies. (**A**) Clade B trimeric wt Env (twt Env), trimeric ΔV2 Env (tΔV2 Env), monomeric wt Env (mwt Env), or monomeric ΔV2 Env (mΔV2 Env) and (**B**) clade A and C trimeric wt Env (twt Env) molecules were incubated with control buffer or increasing concentrations of Tat, and then added to MDDCs at 1∶100 final dilution. Cells were then stained for intracellular Env. Open circles: control isotype mAb; filled circles: anti-integrin mAbs directed against the α5β1, αvβ3 and αvβ5 integrins (10 µg/mL each). Data from the same donor out of 3–8 donors tested are shown. Results are expressed as the percentage of Env-positive cells.

### Production of Recombinant Replication Defective Viruses and Virus Titration

Single-cycle, luciferase-expressing HIV-1 SF162LUC virus was generated by cotransfection of 293T cells with pNL4-3.Luc.R- E- and pCAGGS SF162 gp160 (obtained from Dr. Nathaniel Landau and Drs. L. Stamatatos & C. Cheng-Mayer, respectively, through the NIH AIDS Research and Reference Reagent Program).

**Figure 9:**
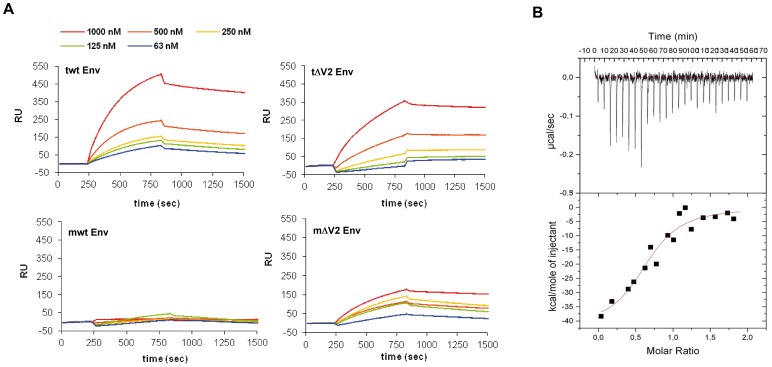
Tat/Env binding kinetics and thermodynamics. (**A**) Binding kinetics of trimeric wt Env (tw Env), trimeric ΔV2 Env (tΔV2 Env), monomeric wt Env (mwt Env) or monomeric ΔV2 Env (mΔV2 Env) to Tat bound to the Biacore chip. The range of concentrations of Env molecules is indicated. (**B**) Tat/Env binding thermodynamics by ITC. Tat was titrated with trimeric wt Env. The top panel shows calorimetric data versus time; the lower panel shows integrated areas normalized to the number of moles of Env subunits injected at each injection step.

**Table 1:** Rate and affinity constants for Tat/Env binding determined by surface plasmon resonance.

	k_on_ (M^−1^s^−1^)	k_off_ (s^−1^)	T_1/2_ (min)	K_d_ (M)
trimeric ΔV2 Env/Tat	2.6×10^3^	5.0×10^−5^	230	2.2×10^−8^
trimeric wt Env/Tat	1.4×10^4^	4.9×10^−4^	23	3.8×10^−8^
monomeric ΔV2 gp120 - Tat	5.9×10^3^	4.9×10^−4^	23	9.1×10^−8^

k_on_, association rate constant; k_off_, dissociation rate constant; T_1/2_, complex dissociation half life; K_d_, binding dissociation constant. No binding was observed with monomeric wt Env.

The single-cycle replication, Tat-independent pseudovirus carrying GFP as gene reporter (VP/SF162) was generated by transient transfection of 293 cells by the calcium phosphate method using pMDLg/RRE, pRSV-Rev (Invitrogen, Life Technologies, Paisley, U.K.), pEF-GFP [49] and the plasmid encoding the envelope protein from HIV (pCAGGS-SF162gp160 obtained from Drs. L. Stamatatos & C. Cheng-Mayer through the NIH AIDS Research and Reference Reagent Program) [50]. This virus does not rely on Tat expression since it does not contain any TAR sequence or Tat coding sequence.

The single-cycle Tat-producing HIV (VSV-G/HIV) was generated by cotrasfection of 293 cells using an envelope deleted HIV plasmid [52] and a VSV-G expression vector [50] as already described [33]. The supernatants harvested from the transfected 293T or 293 cells were collected after 48 h and filtered through 0.45 µm pore-size filters (Millipore, Billerica, MA, USA). Pseudovirus supernatants were assayed for reverse transcriptase (RT) activity [51] to determine virus titers (cpm/mL).

### MDDC Cultures and Infection Experiments

Monocyte-derived dendritic cells (MDDCs) were obtained by culturing monocytes from healthy donors for 6 days in medium containing GM-CSF (500 U/mL; R&D Systems, Minneapolis, USA) and IL-4 (1,000 U/mL; R&D Systems), as previously described [29]. DC maturation was induced by LPS (1 µg/mL) (Sigma-Aldrich, Milano, Italy). MDDC phenotype was assessed by detection of specific surface markers (CD1a, CD14, CD40, HLA-DR, CD83 and CD86) as described [29]. Purity of MDDCs was always ≥99% as assessed by flow cytometry.

For infection experiments with soluble Tat, pseudotyped SF162LUC virus (4.4×10^5^ or 1.2×10^6^ cpm of RT activity/1×10^6^ cells) was incubated with cys_22_ Tat protein at the indicated concentrations or control buffer (PBS - 0.1% BSA) in a 2 or 1 mL volume for 30 min at 37°C prior to infection of immature MDDC cultures. After 2 h at 37°C, cells were extensively washed and cultured for 7 days. Between 3 and 8 days post infection, 3×10^5^ or 6×10^5^ cells, respectively, were lysed and analyzed for luciferase activity by using a Victor luminometer (Perkin-Elmer Life Science, Shelton, CT). Results were expressed in Relative Light Unit (RLU). Experiments were carried out in duplicate.

**Figure 10:**
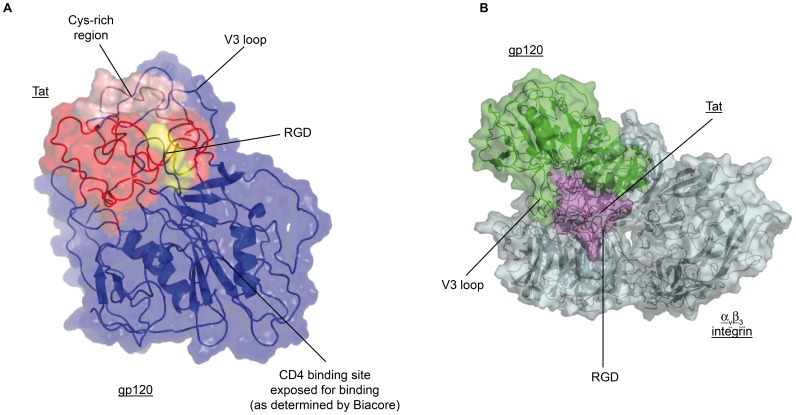
Tat/Env complex and ternary Tat/Env/αvβ3 complex by modeling-docking calculations. (**A**) ribbon representation of the Tat/Env complex showing that the Env CD4 binding site and the RGD domain of Tat are both exposed. Color code: ΔV1-2 Env: blue; Tat: red; Tat-RGD: yellow. (**B**) Surface representation of the ternary Tat/Env/αvβ3 complex. Color code: ΔV1-2 Env: green; Tat: purple; αvβ3 integrin: grey. See experimental procedures for details.

**Figure 11:**
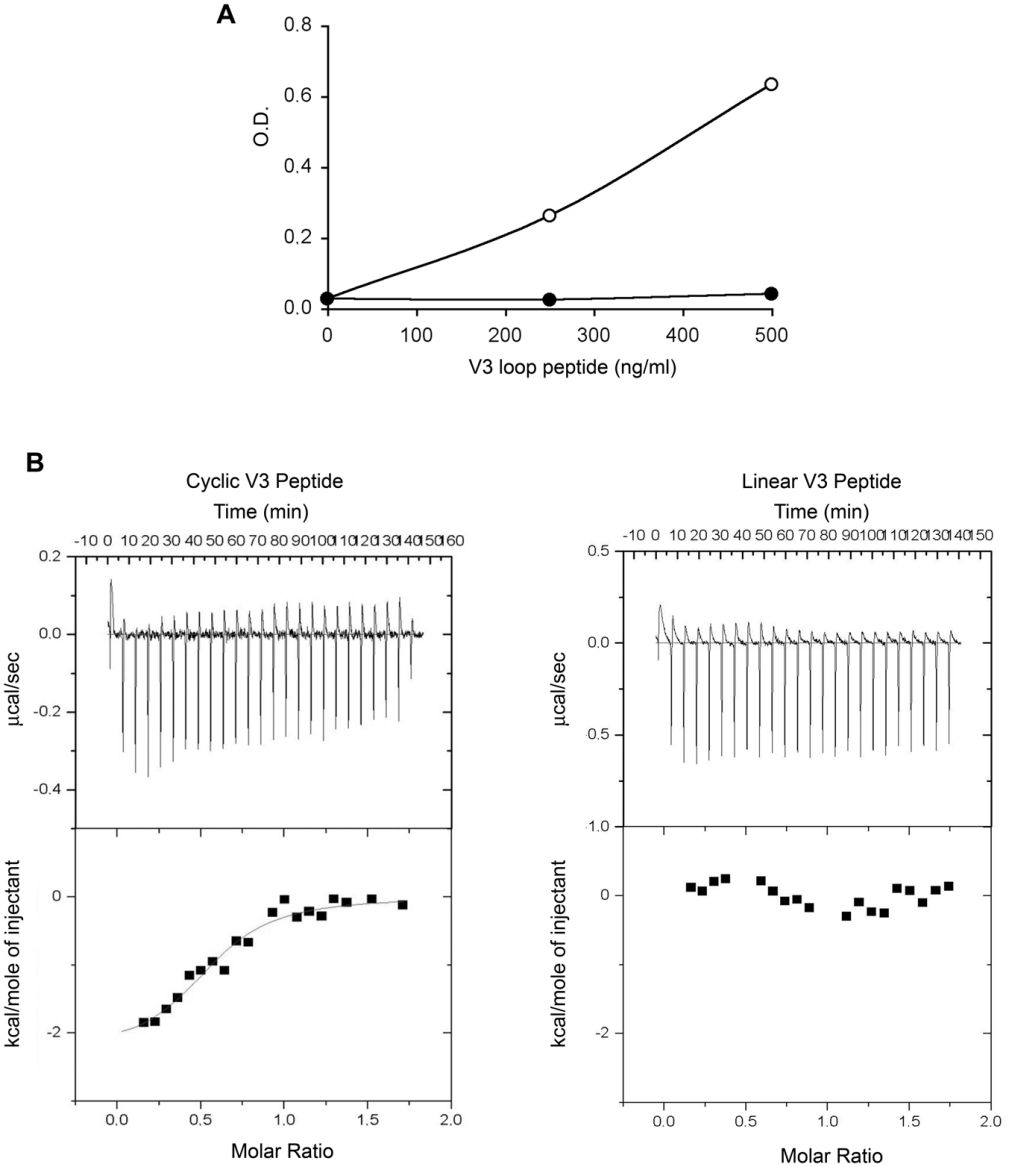
Tat/V3 loop interaction by ELISA and ITC (**A**). Tat interaction with cyclic (open circles) or linear (filled circles) V3 peptides by ELISA. Data are expressed as O.D. corrected for wells coated with the Tat buffer. (**B**) Tat interaction with cyclic (left panels) or linear (right panels) V3 peptides by ITC. Upper panels: calorimetry data versus time; lower panels: integrated areas normalized to the number of moles of V3 loop injected at each injection step.

For infection experiments on adherent Tat, plates were coated with 0.01 or 0.1 µM Tat, 0.5 mL/well, in a 24-wells plate overnight (O/N) at 37°C, washed with 0.5 mL of PBS with Ca/Mg +0.1% BSA and blocked with the same buffer at 37°C for 2 h. MDDCs (1×10^6^) were then seeded on the plates and infected for 2 h at 37°C with HIV SF162 (titer: 1×10^6^ TCID_50_/mL, used at 1∶10 dilution). Infection was evaluated as p24 antigen content by quantitative ELISA (Innotest HIV antigen Mab, Innogenetics, Gent, Belgium), according to the manufacturer’s instructions.

For the co-culture infection experiments, CEMss cells producing Tat (CEMss-Tat) and TZM-bl cells infected or not with the Tat-independent single-cycle VP/SF162 were used. CEMss cells (3×10^5^) were suspended in 0.1 mL of VSV-G/HIV supernatants (7×10^5^ cpm), incubated for 2 h, then washed three times with PBS, resuspended in complete medium and cultured for an additional 24 h. Tat production by CEMss cells was evaluated by immunofluorescence assay. After 24 h, CEMss-Tat cells were co-cultured with TZM-bl cells (3×10^5^) in the presence or absence of VP/SF162 (160,000 cpm). Cells and virus were incubated for 4 h in a small volume at 37°C and then diluted to 0.5 mL with complete medium and cultured in a 24-well plate. After 4 days of co-culture, cells were analyzed for GFP expression by flow cytometry, using a FACSCalibur analyzer (Becton Dickinson). Results were expressed as Mean Fluorescence Intensity (MFI).

**Figure 12:**
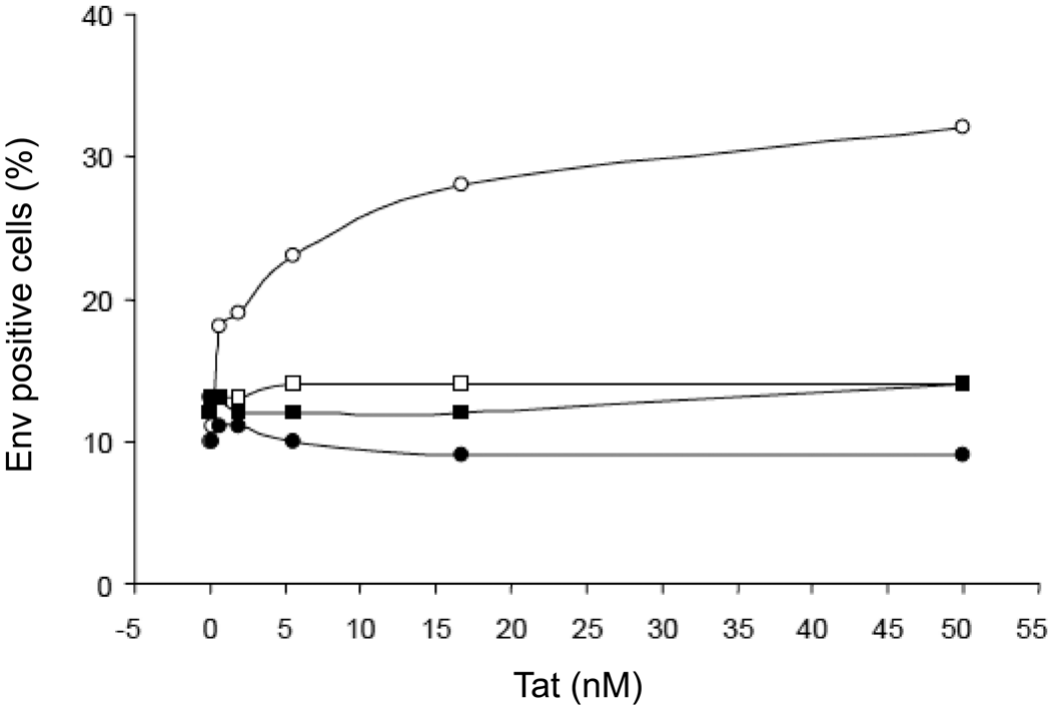
Uptake of trimeric ΔV3 Env by MDDCs and block by anti-integrins mAbs. Uptake of ΔV2 Env or ΔV3 Env pre-incubated with buffer or increasing concentrations of Tat, in the presence or absence of anti-integrin mAbs (10 µg/mL each) or a control isotype mAb (30 µg/mL). Trimeric ΔV2 Env: circles; trimeric ΔV3 Env: squares. Control isotype Ab: open symbols; anti-integrin blocking mAbs combined: filled symbols.

**Figure 13:**
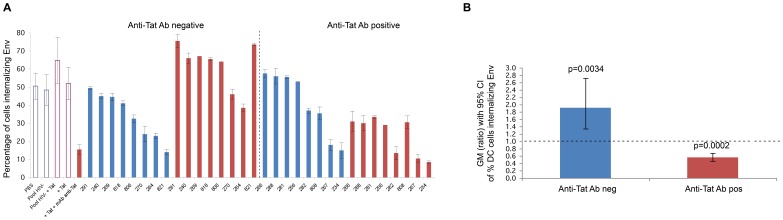
Neutralization of Tat/Env complex entry in MDDCs by sera from HIV-infected individuals. (**A**) Neutralization of trimeric ΔV2 Env entry in MDDCs by sera from HIV-infected subjects in the presence or absence of Tat in anti-Tat Ab negative (n = 8) and anti-Tat Ab positive (n = 8) subjects. The bars represent the percentage of entry of Env alone incubated in buffer (in blue) or with Tat (in red). The percentage of Env-positive cells is shown. Data are expressed as the mean with standard deviation of experiments performed in duplicate. The codes of the anti-Tat Ab negative or positive sera are indicated at the bottom of the bars. (**B**) Geometric mean (GM) of the ratio, with 95% confidence interval (CI) of the percentage of MDDCs internalizing Env in the absence (blue bar) or in the presence (red bar) of Tat in anti-Tat negative (n = 8) and anti-Tat positive (n = 8) subjects. Statistical analysis was performed by the two-tailed Student’s *t*-test.

For experiments of infection transmission to CD4^+^ T cells, MDDCs were seeded on 24-well plates coated with either wt Tat (0.1 µM) or with control buffer, and infected for 2 h at 37°C with HIV SF162 (titer: 10^6^ TCID_50_/mL, used at 1∶10 dilution). The culture supernatants were collected at different time points (3, 7 and 10 days) and added to CD4^+^ T cell blasts previously stimulated O/N with PHA (2×10^6^/well, 24 well/plate) for 2 h at 37°C in the presence of IL-2 (50 IU/mL, Sigma, Italy). CD4^+^ T cells were then collected, extensively washed and plated again with IL-2. The CD4^+^ T cell culture supernatants were collected at 3, 7 and 10 days, and tested for p24 antigen content by ELISA.

**Figure 14:**
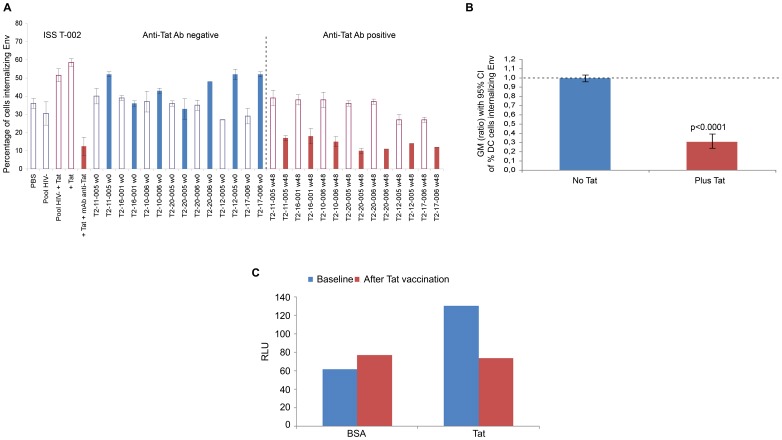
Neutralization of Tat/Env complex entry or infection in MDDCs by sera from Tat-vaccinated HIV-infected individuals. (A) Percentage of MDDCs internalizing Env in the presence of sera from trial subjects (n = 7) at baseline (week 0, anti-Tat Ab negative, in blue) and after Tat vaccination (week 48, anti-Tat Ab positive, in red) in the absence (empty bars) or in the presence (filled bars) of Tat. The codes of the sera are indicated at the bottom of the bars. (B) Geometric mean (GM) of the ratio (week 48 vs baseline), with 95% confidence interval (CI) of the percentage of MDDCs internalizing Env in the presence or in the absence of Tat in trial subjects (n = 7). Statistical analysis was performed by the two-tailed Student’s *t*-test. (C) MDDC infection performed in duplicate for 48 h with pSF162LUC in the presence of coated cys_22_ Tat (0.01 µM) or BSA (control) with sera from a representative trial subject before (baseline, anti-Tat Ab negative) and after Tat vaccination (anti-Tat positive). Sera prior to or after vaccination contained the same anti-Env Ab titers. Data are expressed as RLU.

In blocking experiments of MDDC infection with anti-integrin Abs, MDDCs were pre-incubated with monoclonal Abs (mAbs) directed against the α5β1, αvβ3 and αvβ5 integrins (10 µg/mL each) (Chemicon International, Temecula, CA), or a control isotype mAb (30 µg/mL) (mouse IgG1, BD Biosciences Pharmingen, San Diego, CA), for 30 min at 4°C prior to infection and then experiments continued as described above.

In neutralization experiments of MDCC infection with sera from HIV-infected individuals vaccinated with Tat, pseudotyped SF162LUC virus (5×10^4^ cpm of RT activity/1×10^6^ cells) was incubated on 24-well plates coated with cys_22_ Tat (0.01 µM, 0.5 mL/well) or with BSA for 10 min at room temperature prior to the addition of 1/30 dilution of serum obtained before or after Tat vaccination. The sera had identical anti-Env IgG titers, as assessed by ELISA. After 1 h at 37°C, immature MDDCs were added to the plates (0.5×10^6^ cells/well) and cultured for 48 h. Cells were then lysed and analyzed for luciferase activity and results expressed in RLU. Infection was conducted in duplicate.

**Figure 15:**
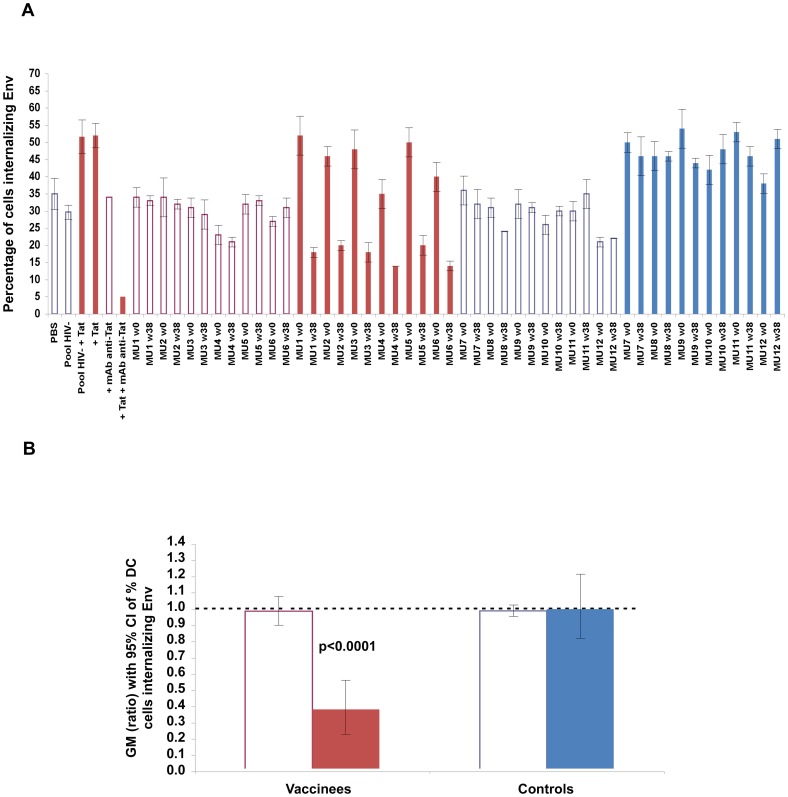
Neutralization of Tat/Env complex entry in MDDCs by sera from Tat/Env-vaccinated monkeys. (**A**) Percentage of MDDCs internalizing Env in the presence of plasma from Tat/Env vaccinated or control monkeys (6 animals/group) before (week 0, anti-Tat Ab negative, in blue) or after vaccination (week 38, anti-Tat Ab positive, in red) in the absence (empty bars) or in the presence (filled bars) of Tat. The codes of the plasma are indicated at the bottom of the bars: MU1-6, vaccinated monkeys; MU7**-**12, control monkeys. (**B**) Geometric mean (GM) of the ratio (week 38 vs baseline), with 95% confidence interval (CI) of the percentage of MDDCs internalizing Env in the presence or in the absence of Tat in vaccinated monkeys (n = 6). Statistical analysis was performed by the two-tailed Student’s *t*-test.

### Production of R5 and X4 Env-VLPs and Tat-mediated VLP Entry by Flow Cytometry and Confocal Microscopy

Fluorescent VLPs were obtained essentially as described [53] by transient transfection of 293T cells by the Lipofectamine-2000 (Invitrogen) method using the pCMVdelta R8.74 HIV-1 packaging vector and the immediate-early CMV promoted vectors expressing NefG3C-GFP and the R5 (HIV-1 BaL) or X4 (HIV-1 HXBc2) envelope proteins, quoted as VLP-R5Env or VLP-X4Env, respectively. As control, VLPs without envelope protein were used (null-VLPs). From 48 to 72 h later, the supernatants were harvested, clarified, and concentrated by ultracentrifugation on 20% sucrose cushion at 100,000 g, for 2 h at 4°C. VPLs were tested by western blot using anti-HIV-1 Gag (clone AG3.0), anti-HIV-1 Nef (clone ARP 444) and the HT-3 anti-HIV-1 Env mAb (AIDS Research and Reference Reagent Program). VLP preparations were titrated by both measuring HIV-1 CAp24 contents by quantitative ELISA (Innogenetics) and RT assay.

For VLP entry experiments, after pre-incubation with different concentrations of Tat or control buffer for 1 h at 4°C, VLPs were added to immature MDDCs (2×10^4^) and spinoculated at 150 g for 30 min at room temperature. Cells were incubated for 4 h at 37°C, then treated for 10 min with trypsin at 37°C to remove residual cell membrane bound VLPs, centrifuged, fixed and analyzed by flow cytometry, fluorescence microscope, and confocal microscope. Results are express as the percentage of fluorescent cells.

For confocal microscopy analysis, VLP challenged MDCCs were fixed in buffered formaldehyde (2% v/v). Phase-contrast and fluorescence images were recorded with an Olympus IX-81 device.

In integrin blocking experiments, prior to the addition of VLP-R5Env, MDDCs were incubated at 4°C for 2 h in the presence of either mAbs directed against the α5β1, αvβ3 and αvβ5 integrins combined (10 µg/mL each) or a control isotype mAb. VLPs were then added and the experiments continued as described above.

**Figure 16:**
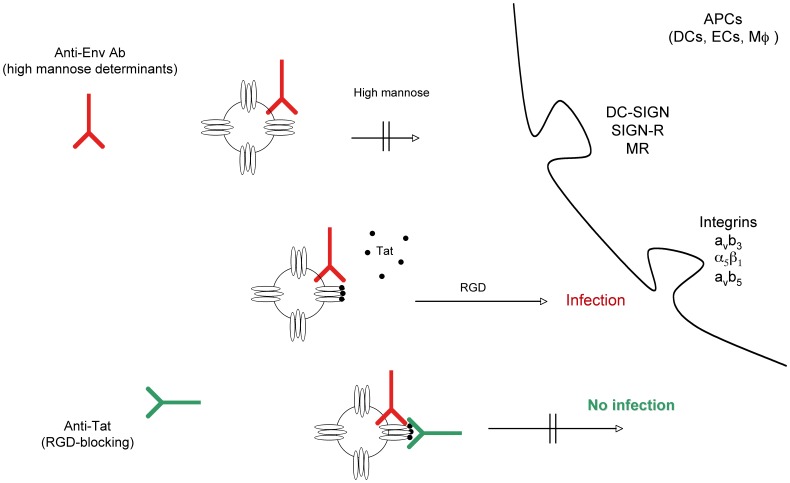
Outcome of DC infection in the absence or presence of Tat, anti-Env and/or anti-Tat antibodies. Tat redirects HIV to RGD-binding integrins evading neutralization by anti-Env Abs and both anti-Env and anti-Tat Abs are required to block infection. Extracellular Tat released by infected neighbor cells binds to trimeric Env on HIV, decreasing recognition of C-type lectin receptors and promoting engagement of RGD-binding integrins, which are expressed by inflammatory DCs, macrophages and endothelial cells (ECs) present at the site of infection. As a result, virions escape neutralization by anti-Env Abs directed against high mannose determinants and enters target cells upon binding to RGD-binding integrins. Anti-Tat Abs neutralize this binding, preventing virus entry through RGD-binding integrins. DC-SIGN: Dendritic Cell-Specific Intercellular adhesion molecule-3-Grabbing Non-integrin; SIGN-R: homologue of DC-SIGN present on ECs; MR: mannose receptor.

### Immuno-scanning Electron Microscopy Analysis of HIV Bound to Tat-coated Latex Beads

Latex beads (1×10^9^, mean diameter 2.4 µm) (Interfacial Dynamics Corporation, Portland, USA) were incubated for 1 h at room temperature with 10 µg of Tat or control buffer, washed three times with 30 mM Na_2_CO_3_, 70 mM NaHCO_3_, (pH 9.5), and incubated for 1 h at 37°C with either concentrated HIV (HXBc2) supernatants or with fresh medium. Beads were then washed three times with PBS-0.1% BSA and incubated for 45 min at 37°C with 1∶100 dilution of anti-gp120 rabbit Abs (Intracel Corporation, Frederick, MD) in the same buffer. After incubation, beads were washed with control buffer and then incubated with 1∶50 dilution of 5 nm gold-conjugated anti-rabbit IgG Abs (Sigma Chemical Company, St. Louis, MO) in the same buffer. After 45 min at 37°C, the beads were washed with 0.2 M cacodylate buffer and then fixed with 2.5% glutaraldehyde in cacodylate buffer for 15 min at room temperature. After washing with distilled water, gold particle labelling was amplified with silver enhancement solution (Sigma Chemical Company) according to the manufacturer’s instructions. For both secondary electrons and backscattered electron observations, samples were transferred on glass coverslips, air-dried, and carbon-coated, and examined with a Cambridge Stereoscan 360 scanning electron microscope.

### Tat and Tat/Env Entry into MDDCs and Blocking by Anti-integrins mAbs

Increasing concentrations of Tat (0, 2, 6, 18, 54, 162, 486 nM) were added to MDDCs (2×10^5^ cells/mL), and after 10 min incubation, cells were washed with cold medium and treated for 5 min at 37°C with trypsin-EDTA (Life Technologies, Paisley, UK) to remove cell-surface bound protein. After fixation and permeabilization (Perm Solution, BD Biosciences), MDDCs were stained with an affinity-purified rabbit polyclonal anti-Tat IgG Abs (Diatheva, Fano, Italy) or rabbit IgG control Abs (ICN Biomedicals, Opera, Italy), followed by FITC-conjugated anti-rabbit Ig (Pierce, Rockford, IL), as described [29]. Fluorescence was analyzed by flow cytometry, and results were expressed as the percentage of Tat positive cells as compared to isotype Ab stained samples. The values from non-permeabilized cells were subtracted.

For Env uptake experiments, a fixed concentration of monomeric and trimeric wt or ΔV2 Env molecules from clade B, A, or C (3.52 µM in protein subunit) was incubated at 25°C in degassed PBS alone or in the presence of increasing concentrations (0.2 – 48.6 µM) of Tat for 10 min. The protein samples were then added to MDDCs (2×10^5^ cells/ml) at a 1∶100 final dilution. Cells were washed with cold medium and treated as described above for Tat uptake experiments, and stained with an anti-gp120 (NeoMPS Strasbourg, France) or rabbit IgG control Abs, followed by FITC-conjugated anti-rabbit Ig (Pierce). Results were expressed as described above.

In integrin blocking experiments, prior to the addition of the proteins, MDDCs were cultured at 4°C for 2 h in the presence of mAbs directed against the α5β1, αvβ3 and αvβ5 integrins alone or combined (10 µg/mL each), or with a control isotype mAb. Results were expressed as described above.

### Blocking of Trimeric ΔV2 Env Entry or Tat/ΔV2 Env entry in MDDCs by Anti-DC-SIGN and Anti-integrin mAbs

MDDCs (2×10^5^ cells/mL) were incubated for 60 min in the presence of 20 or 50 µg/mL of the anti-CD209 mAbs (DC-SIGN) (Beckman Coulter), anti-integrins mAbs (Chemicon International), an IgG1 control isotype or nil, washed twice with PBS and then incubated for 10 min with 35 or 70 nM trimeric ΔV2 Env (SF162) prior to Env intracellular staining and flow cytometry analysis.

In the experiments of trimeric ΔV2 Env/Tat complex entry in MDDCs, cells were incubated, as previously described, in the presence of 50 µg/mL of the anti-CD209 mAb and anti-integrins mAbs or a control isotype mAb, and then incubated for 10 min with trimeric ΔV2 Env (70 nM) previously complexed with Tat at different concentrations (50, 16.67 and 5.56 nM) prior to Env intracellular staining and flow cytometry analysis. In both settings, results were expressed as the percentage of Env-positive cells.

### Blocking of Tat/Env Complex Binding to PBL by an Anti-CD4 mAb

Peripheral blood lymphocytes (PBLs) were isolated from PBMCs upon 2 h plastic adherence at 37°C to remove the majority of monocytes. PBLs (2×10^5^ cells/100 µL/tube) were pre-incubated for 10 min at room temperature with nil or an anti-CD4 mAb (clone Q4120, Progenics) and then incubated for 1 h at room temperature with trimeric wt Env (250 nM) or wt Env which had been pre-incubated for 10 min with the indicated amounts of Tat. After 30 min, PBLs were extensively washed, stained with an anti-gp120 mAb FITC (US Biological, H6003-34J3) for 30 min at 4°C, washed, fixed with paraformaldeyde and analyzed by flow cytometry. Results were expressed as Env-binding positive cells.

### Surface Plasmon Resonance (SPR) Assay

SPR assays were performed using a BIACORE 3000 (Biacore AB, Uppsala, Sweden). Tat was immobilized using amine coupling onto the CM3 sensor chip. As a positive control for the quality and activity of the Env molecules, four domains soluble CD4 was immobilized using amine-coupling reaction onto the same sensor chip. The Env to Tat association was assessed by flowing different concentrations of Env (5–1,000 nM) in PBS buffer, pH 7.4, with 0.2% Tween 20, 300 mM NaCl, and 5 mg/mL dextran. The non-specific association of Env to Tat was determined using a control surface without any ligand. Soluble CD4, an anti-CD4 mAb, and recombinant spike protein of the SARS virus were used as negative controls. Sensor data were prepared for kinetics analysis by subtracting binding responses collected from a blank reference surface. The association and dissociation phase data were fitted simultaneously to a single-site model by using the nonlinear data analysis program Biaevaluation 4.01 (Biacore AB, Uppsala, Sweden).

### Isothermal Titration Calorimetry

Isothermal titration calorimetry was performed on a VP-ITC (Microcal LLC, Northampton, MA). Proteins were suspended in PBS pH 7.4 and Tat (1.25 to 4.4 µM) was loaded into the cell with trimeric Env (12.5-to 47.0-µM in protein subunit) in the syringe. All runs were adjusted for heat of dilution and mixing. Binding isotherms were auto-fitted to a baseline using the Origin® software. For V3 loop, recombinant Tat and cyclic (heat-to-tail) or linear V3 loop peptides lacking cysteins were suspended in PBS (pH 7.4) containing 5 mM DTT. Twenty-eight µM Tat was titrated at 20°C with a 260 µM peptide solution. The heat of dilution of the peptide was subtracted and the binding isotherm determined with the Origin software.

### Modeling and Docking Calculations

Structural models of (i) the Tat protein (BH10) and (ii) the gp120 ΔV1-2 domain of Env (SF162) in the CD4-free form, were generated with the Modeller8 program [54] starting from the available structure coordinates (see below) (Table S2), and optimized through energy minimization with AMBER8 [55]. To map the conformational variability of the Tat protein, three different models were built on the basis of the three available NMR structures [56], [57] that, in spite of their high pair-wise sequence identity (>77%), show different conformations (the average RMSD among the three structures, calculated on the average NMR structures, is 5.9 Å). The core of the CD4-free form of the gp120 Env protein was modeled on the ΔV1-2-3 SIV gp120 structure [58] and the V3 loop added using as template the V3 loop present in the CD4-bound ΔV1-2 HIV-1 gp120 structure [59]. To sample the range of accessible conformations for the V3 loop, a 10 ns long molecular dynamics simulation was performed on the gp120 Env ΔV1-2 structural model (details in Table S3), and an ensemble of 50 different Env conformations was selected and used for subsequent Tat/Env docking calculations. Models of the Tat/Env complex were at first calculated with the BIGGER [60] and ClusPro programs [61]. The interaction surfaces resulting from these calculations, all of which consistently included the V3 loop, were then used as input for Ambiguous-restraints Driven Docking calculations of the Tat/Env complex, with the HADDOCK program [62] (Table S4). This program was also used for modeling the complex between integrin αvβ3, whose structure is available in the pdb (pdb identifier: 1L5G) [63] and the energy minimized structures of Tat. For this complex, both unambiguous and ambiguous restraints were used (Table S5), the former being derived from the structure of an adduct between integrin and a cyclic peptide containing the RGD sequence [63]. The ternary complex Tat/Env (ΔV1-2) was derived by superimposing Tat in the binary complexes Tat/Env (ΔV1-2) and Tat in the binary complex Tat/integrin αvβ3 and generating a structural model which involves Tat, Env (ΔV1-2) and integrin αvβ3_._

### ELISA for Binding of V3 Peptides to the Tat Protein

Microtiter ELISA plates were coated with 100 ng per well of Tat in a 100 µL volume of Dulbecco’s PBS containing Ca^2+^ and Mg^2+^ for 1 h at 37°C. Plates were washed and blocked with control buffer for 2 h at room temperature. Wells were then incubated with different concentrations of a cyclic or a linear V3 peptide lacking cysteins in blocking buffer for 1 h at 37°C. After washing, plates were incubated with a primary Ab (rabbit anti-V3 serum 1∶1,000) for 1 h at 37°C, incubated with anti-rabbit-peroxidase Ab (SIGMA) (1∶2,000) for 1 h at 37°C and developed with ABTS substrate (Roche). Results were expressed in optical density (OD).

### Neutralization of Tat/Env Entry in MDDCs by Anti-Tat Ab Negative or Ab Positive Sera from HIV-infected Asymptomatic or Tat-vaccinated Subjects or from Tat/Env-vaccinated Monkeys

For experiments with human anti-HIV sera, sera containing or not anti-Tat Abs from asymptomatic HIV-infected individuals or from vaccinees (at baseline or 48 weeks after the first immunization) of ISS T-002 therapeutic trial were used.

The ISS T-002 (ClinicalTrials.gov NCT00751595) is a randomized, open-label, phase II clinical trial with the biologically active HIV-1 Tat protein conducted in 168 HIV-1-infected HAART-treated anti-Tat Ab negative subjects, with undetectable levels of plasma viremia (<50 copies/mL) since the last 6 months prior to enrollment, CD4^+^ T cell number ≥200 cells/µL and known pre-HAART CD4 nadir [13]. The ISS T-002 clinical trial was approved by the competent authority (General Director of the Coordinator Clinical Center, Policlinico of Modena, Modena) and by the Ethics Committees of each participating clinical center (Policlinico of Modena, Modena; Arcispedale S. Anna, Ferrara; Istituti Fiosterapici Ospitalieri San Gallicano, Rome; Policlinico of Bari, Bari; Ospedale S.M. Goretti Latina; Fondazione S. Raffaele, Milan; Ospedale S. Maria Annunziata Florence; Ospedale Luigi Sacco, Milan; Spedali Civili, Brescia; Ospedale A. di Savoia, Turin). All subjects signed an informed consent at study entry.

Sera were diluted 1∶30 in PBS and incubated for 60 min at 37°C with trimeric ΔV2 Env (0.4 mM) premixed for 10 min at 25°C with Tat (0.4 mM) or degassed PBS. The samples were then added to MDDCs (2×10^5^ cells/mL) to a 1∶5 final dilution. Cells were then stained for intracellular Env and results expressed as the percentage of Env positive cells. In these experiments Env was added to the cells to a 0.08 mM final concentration, which resulted in a higher percentage of positive cells for determining the degree of inhibition of Env uptake by the sera. Due to the high cell saturation level by Env, the degree of stimulation of Env uptake by Tat was correspondingly lower. A pool of sera from six healthy donors and a mAb against Tat (mAb 2A4.1, obtained from Dr Jon Karn through the AIDS Research and Reference Reagent Program, Division of AIDS, NIAID, NIH) were used as controls. To assess the capability of mAb 2A4.1 to interfere with Tat uptake by MDDCs, Tat was pre-incubated or not with molar excesses of the mAb prior to the addition to MDDCs and then Tat uptake determined by intracellular staining in flow cytometry.

For experiments with monkeys’ plasma, samples from Tat/Env vaccinated or control monkeys at baseline (week 0) or after immunization (week 38) were used. To assess the neutralization of the Tat/Env complex entry in MDDCs, the same experimental procedures described above for the human sera were used.

### Statistical Methods

Viral RNA and proviral DNA in blood or tissues were compared between vaccinated monkeys and controls by the one-tailed Wilcoxon rank sum test. Percentage of MDDCs internalizing Env in the presence or absence of Tat in HIV infected anti-Tat Ab negative or anti-Tat Ab positive subjects, as well as changes from baseline of the percentage of MDDCs internalizing Env in Tat-vaccinated trial subjects (ISS T-002) or in Tat/Env-vaccinated monkeys were assessed by the two-tailed Student’s *t*-test (log_10_ transformation was performed to normalize the data distribution). Statistical analyses were carried out at the 0.05 significance level, using SAS^®^ software, version 9.2 (SAS Institute, Cary, NC).

## Results

### Co-immunization of Cynomolgus Macaques with HIV-1 Tat and Env Proteins Blocks Systemic Spreading of an R5 Challenge Virus

To evaluate the effect of mucosal immunization with the Tat and the oligomeric ΔV2 Env protein combination, cynomolgus macaques were vaccinated twice intranasally with HIV-1 Tat and Env, given together with the LT-K63 mucosal adjuvant, and then boosted subcutaneously with Tat plus Env in Alum. Controls received only the adjuvants by the same routes (Table S1). Macaques were then challenged intrarectally with 70 MID_50_ of the R5-tropic SHIV_SF162P4cy_ using a virus stock adapted to cynomolgus macaques and titrated in vivo [41]. No infection or a statistically significant reduction of viral loads and proviral DNA were observed in the vaccinated monkeys as compared to controls (Fig. 1 A–B and Table S6). Of note, proviral load in the inguinal lymph nodes was significantly lower in vaccinated monkeys as compared to controls, whereas it did not differ significantly in rectal biopsies (Fig. 1C). Thus, mucosal vaccination with the Env and Tat protein combination leads to an effective containment of viral infection at the portal of entry, with block of virus dissemination to lymph nodes following a very high rectal challenge dose.

### Extracellular Tat Enhances Infection of Monocyte-derived Dendritic Cells (MDDCs) by Promoting Virus Entry

Since DCs are key cells at the portal of entry of HIV, and Tat is known to act on them by binding to their RGD-integrin receptors, we investigated whether Tat affects their susceptibility to virus infection. To this goal, a single-cycle luciferase-expressing R5 HIV-1 (SF162LUC), pre-incubated or not with the transactivation-silent cys_22_ Tat protein (Fig. S1), was added to MDDCs from different donors. The cys_22_ Tat was used to dissect effects of Tat on virus infection other than its transactivation activity. The cys_22_ Tat enhanced HIV infection in MDDCs to a different extent (ranging from 6.6 to 1.6 fold increase) according to the donor (Fig. 2). Thus, Tat increases HIV infectivity in DCs by a novel, transactivation-independent mechanism(s), suggesting that Tat can increase virus entry.

To investigate the effect of extracellular Tat on HIV entry, fluorescent VLPs, displaying on their surface native trimeric HIV-1 Env from either the R5-tropic BaL or the X4-tropic HXBc2 strain (Env-VLPs), were pre-incubated with or without increasing concentrations of the cys_22_ Tat protein and then added to MDDCs. VLPs without Env (null-VLPs) were used as control. As shown in Fig. 3A and B, the proportion of MDDCs positive for Env-VLPs from both viruses markedly increased in the presence of Tat. Confocal microscopy confirmed that Env-VLPs were internalized by the cells (Fig. 3C). In contrast, the effect of Tat on the entry of null-VLPs was negligible, ruling out an effect of Tat on the endocytic pathway. In addition, Tat did not increase Env-VLP internalization when was given at the same time of VLP addition to MDDCs (data not shown), suggesting that Tat must be already bound to Env-VLPs to observe internalization. This indicated that Tat increases virus entry and suggested that the increased VLP entry is receptor-mediated and dependent on Tat/Env binding interactions.

### Tat Released by Producing T cells Increases Entry and Infection of a Replication-incompetent and Tat-independent SF162-pseudotyped Virus

Tat is released by acutely infected cells and extracellular Tat is present in tissues from infected subjects [14]–[21]. In order to determine whether extracellular Tat produced by neighbor cells could affect the entry of virus particles and infection independently of its transactivating activity, a co-culture system was used in which CEMss-Tat expressing cells (which do not express CCR5) and TZM-bl cells, which are CCR5^+^, were co-cultured in the presence or absence of a single-cycle, Tat-independent and GFP expressing SF162 (VP/SF162) [64], as described in materials and methods. As shown in Fig. 4, the MFI of GFP positive TZM-bl cells was greatly enhanced only in the presence of CEMss-Tat cells. Since CEMss cells do not express detectable levels of CCR5, VP/SF162 could only infect the CCR5^+^ TZM-bl. Thus, Tat released by infected cells promotes the entry of virions into neighbor cells, favoring spreading and dissemination of the infection.

### Extracellular Tat Coated on Beads Binds HIV Virus Particles and Adherent Tat Increases Productive Infection of MDDCs, which Efficiently Transmit the Virus to T cells

Once released, Tat binds the HSPG present on the ECM and on the cell surface. Tat present in tissues is mostly bound whereas its soluble form is estimated to be lower [14], [15], [21]. In addition, adherent Tat increases infection efficiency of T cells by both X4 and R5 HIV strains in a transactivation-independent manner [33].

To investigate whether adherent Tat is capable to bind HIV virions, latex beads were coated with the Tat protein or its buffer, and virus (HXBc2) added. After extensive washes, beads were analyzed by immuno-Scanning Electron Microscopy using an anti-gp-120 mAb. The backscattered electron imaging revealed that HIV particles were bound only to Tat-coated beads, whereas no virus was found on beads pre-incubated with the Tat buffer containing BSA (Fig. 5A). Thus, immobilized Tat binds and retains very efficiently HIV virus particles.

To determine whether adherent Tat, which mimicks the biologically relevant form found in tissues, is capable of mediating on MDDCs the effect observed with the soluble protein, infection experiments were carried out using non tissue-culture plates in which wells had been coated with Tat or BSA (control). As shown in Fig. 5B, MDDC infection with SF162 HIV onto Tat-coated wells resulted in a several fold increase of productive cell infection, as determined by measuring p24 content in the cell supernatants, as compared to cells in wells coated with BSA. Thus, as the soluble form, adherent Tat promotes HIV entry and productive infection of MDDCs. Moreover, the viral progeny from these cells was highly infectious, as determined by the transmission of a productive infection to T cells with supernatants of MDCCs infected onto Tat-coated wells as compared to BSA-coated wells (Fig. 5C).

This suggested that extracellular Tat present on virus particles [33] or released by infected cells greatly contributes to the acquisition and spreading of HIV infection. This occurs in a transactivation-independent manner and by promoting virus entry and productive infection of DCs. On note, infection of DCs would be further amplified by endogenous Tat produced during infection.

### Antibodies to RGD-binding Integrins Neutralize Tat-mediated Entry and Infection of MDDCs

Extracellular Tat targets and binds through its RGD region the integrins α5β1, αvβ3 and αvβ5 expressed by DCs and other cells of the reticular-endothelial system [22]–[29], and co-stains with these integrins in tissues of HIV-infected individuals [14]–[20].

To elucidate the contribution of RGD-binding integrins in the enhancing effect of Tat on MDDC infection, experiments were performed with either soluble cys_22_ Tat or coated wt Tat with MDDCs pre-incubated with a mixture of mAbs directed against the α5β1, αvβ3 and αvβ5 integrins. BSA was used as control. As shown in Fig. 6, anti-integrins mAbs efficiently neutralized the enhancement of infection mediated by both soluble (Fig. 6A) or adherent (Fig. 6B) Tat, indicating that they are involved in the Tat-mediated virus entry and productive infection of DCs.

### Tat Enters MDDCs through RGD-binding Integrins and Targets Oligomeric Env to these Receptors

Since Tat enters very efficiently into MDDCs [29], experiments were performed to determine whether this occurs through RGD-binding integrins, and whether this could mediate entry of Tat-bound virus particles or trimeric Env molecules.

As shown in Fig. 7, Tat entered very efficiently MDDCs, and entry of both wt Tat (Fig. 7A) and cys_22_ Tat (Fig. 7B) was completely blocked by a combination of mAbs directed against these RGD-binding integrins. As shown in Fig. 7C, the anti-integrin mAbs inhibited also Tat-mediated entry of Env-VLPs into MDDCs. This suggested that Tat enhances HIV entry into MDDCs via the integrin endocytic pathway after forming a complex with Env.

This was addressed by intracellular staining upon incubation of MDDCs with Tat or Env proteins alone or combined. As shown in Fig. S2, Tat entry into MDDCs was not affected by any of the Env proteins (oligomeric or monomeric) used, while it was completely inhibited by the anti-integrin mAbs, confirming that binding of the RGD region of Tat to integrins is required for Tat entry and that this is not affected by the presence of Env.

With regard to Env, in the absence of Tat, all Env molecules efficiently entered MDDCs upon binding to C-type lectin mannose-binding receptors, as indicated by the inhibition of Env entry by a mAb blocking DC-SIGN (dendritic cell-specific ICAM-3 grabbing non-integrin) (Fig. S3). Notably, in the absence of Tat, the entry of Env molecules was not affected by the anti-integrin mAbs, confirming that they are not involved in the entry of Env (Fig. 8A, S2 and S3). However, when Env molecules were pre-incubated with increasing concentrations of Tat, the entry of Env augmented in a Tat dose-dependent manner reaching rapidly a plateau in the nanomolar range (Fig. 8A and S3C). This entry of Env was efficiently blocked by the anti-integrin mAbs (Fig. 8A and S3C). In particular, the proportion of Tat-mediated, integrin-dependent Env entry was most pronounced with the trimeric ΔV2 Env as compared to trimeric wt Env or the monomeric ΔV2 Env, and lowest with the monomeric wt Env (Fig. 8A). Of note, the effects of Tat on trimeric Env were also observed with wt Envs from both clade A and C (Fig. 8B). Finally, blocking of both integrin and DC-SIGN receptors did not completely block the entry of Env (Fig. S3C), suggesting either incomplete blocking of the above receptors or, more likely, the existence of additional minor binding and entry pathways. These data suggest a specific interaction of Tat with Env determinants, which appear to be better exposed by the trimeric Env, especially upon deletion of the V2 loop, and to be conserved among different HIV clades.

### Tat Forms a Stable High-affinity Complex with Trimeric Env by Binding gp120 Subunits

Surface plasmon resonance (SPR) was then used to gain more insights into the Tat/Env interaction. As shown in Fig. 9 and Table 1, the binding profile and the rate constants indicated the formation of stable complexes for both the trimeric wt (half-life: 23 min) (Fig. 9A, upper left panel) and trimeric ΔV2 Env (half-life: 230 min) (Fig. 9A, upper right panel) as well as for monomeric ΔV2 Env (half-life: 23 min) (Fig. 9A, bottom right panel), but not for monomeric wt Env, which did not appear to bind Tat in these assays (Fig. 9A, bottom left panel). Thus, deletion of the V2 loop increases the affinity and/or stability of the complex in both trimeric Env and monomeric gp120 (Table 1), explaining the differences observed in the DC uptake experiments.

To determine the number of Tat binding sites on gp120, isothermal titration calorimetry (ITC) was performed with trimeric wt Env. As shown in Fig. 9B, the binding isotherm indicated a 1∶1 stoichiometry, with one molecule of Tat bound per gp120 subunit in the trimer. In substantial agreement with SPR measurements, a K_d_ of 111±43 nM was determined.

### Tat Binds Env Domains Overlapping Both the V3 Loop and the CCR5-binding Sites

To localize the region of Env recognized by Tat, structural models for the interaction of the two proteins were determined by docking calculations. To model the two partner proteins, templates derived from experimental structures were used. For monomeric gp120 Env ΔV1-2 a structural model was derived from the X-ray structures of both an unliganded (not CD4-bound) simian immunodeficiency virus gp120 and a V3 loop from a liganded HIV gp120 (see experimental procedures for details); for the Tat structural model all the available solution structures were used after energy minimization and molecular dynamics. For the docking calculations it resulted that the interaction region of Env with Tat consistently included the V3 loop (Fig. 10 and Fig. S4). In particular, this interaction region included both sides of the V3 stem loop, which is one of the two main CCR5-binding regions of Env, suggesting that Tat recognizes a bent V3 loop conformation (Fig. 10A and Fig. S4). In these models the interaction occurred with Tat residues spanning the Cys-rich region (aa 22–37). Importantly, the C-terminal part of Tat containing the RGD sequence remained free as needed for integrin binding (Fig. 10 and Fig. S5 and S6), as shown by the structural model of a ternary complex of Tat and Env with the αvβ3 integrin (Fig. 10B and Fig. S5). Further, in these models the CD4-binding region of Env was also free and not buried into the complex (Fig. 10), as confirmed by blocking the CD4 binding of the Tat/Env complex to peripheral blood lymphocytes (PBL) by a mAb against CD4 (Fig. S7).

The validity of these complex models was then verified by ELISA and ITC experiments using a cyclic or a linear V3 loop peptide. As shown in Fig. 11, the cyclic but not the linear peptide bound Tat both in ELISA (Fig. 11A) and ITC (Fig. 11B). In addition, the ITC binding isotherm indicated a 1∶1 stoichiometry as for the interaction with trimeric Env. This confirmed a direct interaction between Tat and the V3 loop in a bent conformation.

The role of the V3 loop in the Tat/Env interaction was further analyzed by using a trimeric Env carrying a deletion in the V3 loop (trimeric ΔV3 Env) and by comparing its uptake by MDDCs with that of trimeric ΔV2 Env, in the presence or absence of the anti-integrin mAbs. The internalization of ΔV3 Env was not enhanced by Tat, nor was affected by the anti-integrin mAbs (Fig. 12). Thus, the V3 loop is necessary for the formation of a stable interaction of Env with Tat.

Modeling-docking calculations also identified additional interactions in the inner domain of Env in proximity to the outer domain and the other CCR5 binding region of Env (Fig. S8), suggesting that Tat interacts and/or modifies both CCR5-binding regions of Env. In fact, a lower K_d_ was found for Tat interaction with the cyclic V3 loop peptide as compared to the whole Env (Fig. 9A and 11B), and an increased stability of the complex was detected upon V2 deletion, which is known to unmask this co-receptor binding region of Env (Fig. 9A upper right panel). Thus, Tat appears to bind and/or overlap to both CCR5 binding regions of Env, and, upon these interactions, to induce conformational changes in Env leading to exposure of new epitopes.

### Anti-Tat Abs are Essential to Neutralize the Tat-mediated Env Entry and Virus Infection of MDDCs and to Restore and Enhance Neutralization by HIV Sera

The previous results indicated that Tat directs virus particles to the RGD-binding integrin endocytic pathway via a mechanism that is independent of Env and DC-SIGN-mediated virus entry, diverting the virus into a Tat-driven and integrin-mediated pathway**,** which is highly efficient for infection of antigen-presenting cells such as DCs.

By directing HIV to RGD binding integrins and/or by shielding Env, Tat may divert Ab responses blocking the Env and the DC-SIGN entry pathway, which is believed to play a key role in T cell *trans*-infection. To test this hypothesis, sera from asymptomatic HIV infected individuals, positive for Abs against Env and negative or positive for Abs against Tat, were used in Tat/Env DC entry experiments. Sera were mixed with oligomeric ΔV2 Env in the presence or absence of Tat prior to the addition to MDDCs. In the absence of Tat, sera showed a variable level of inhibition of Env entry in MDDCs (Fig. 13A) indicating that Abs against Env can partially inhibit the interaction of Env with DC-SIGN. However, sera from all subjects negative for anti-Tat Abs became almost totally ineffective at inhibiting Env entry into MDDCs in the presence of Tat (Fig. 13A). In contrast, sera from all subjects also positive for anti-Tat Abs remained neutralization effective in the absence of Tat, and their effectiveness was greatly increased or induced *ex novo* by Tat (Fig. 13A). Thus, in the presence of Tat, anti-Env Ab positive but anti-Tat Ab negative sera lose their neutralizing effect on virus entry, while the presence of both anti-Env and anti-Tat Abs restores and significantly increases HIV neutralization (Fig. 13B).

To determine whether Tat vaccination could induce anti-Tat Abs capable of blocking Tat/Env entry and infection of MDDCs, sera from HIV infected HAART-treated patients from a phase II Tat therapeutic trial (ClinicalTrials.gov NCT00751595) [13] were tested prior to and after vaccination. As shown in Fig. 14, Env entry into MDDCs was partially blocked by the anti-Env Ab positive and anti-Tat Ab negative sera collected at baseline from the vaccinees. However, in the presence of Tat, neutralization of Env entry by these sera was lost. In contrast, after Tat vaccination, sera inhibited the effect of Tat on Env entry and further increased neutralization (Fig. 14A), in a statistically significant manner (Fig. 14B). Similar results were obtained in MDDC infection in the presence of Tat (Fig. 14C), which reproduced the same pattern as that observed for the Tat/Env entry assays, confirming that Tat vaccination can induce anti-Tat Abs capable of blocking both entry and infection mediated by Tat. In particular, sera abolished the enhancement effect of Tat on DC infection which diminished to the levels observed with BSA (negative control). In addition, comparable results on cell infection were obtained with purified anti-Tat rabbit polyclonal antibodies.

Finally, similar or identical results were also obtained with plasma from Tat/Env vaccinated monkeys (Fig. 15), in that plasma from vaccinated animals blocked the enhancement of Tat-mediated entry of Env and further neutralized Env uptake, as compared to their baseline or to control animals.

Thus, anti-Tat Abs are essential to neutralize the Tat and Env complex entry and virus infection, and the presence of both anti-Env and anti-Tat Abs, as occurring in Tat vaccinated HIV infected patients or in Tat/Env vaccinated monkeys, is required to block HIV entry and infection (Fig. 16).

## Discussion

In the present study, we found that mucosal vaccination with the combination of Tat and Env proteins protected non-human primates against an intrarectal challenge with a high dose of an R5-SHIV. In particular, a highly significant containment of the virus at the portal of entry was found in vaccinated monkeys as compared to controls, with block of virus dissemination to lymph nodes, the primary site for virus amplification and establishment of the virus reservoir. Following this, we found that extracellular Tat**,** both soluble and adherent, was able to increase both R5 and X4 HIV entry and infection of DCs, a major HIV cell target present at the mucosal portal of entry. This action of Tat is transactivation-independent, and is due to the formation of a molecular complex between Tat and trimeric Env. By increasing entry of trimeric Env, Tat bound to virus particles increases virus infection of DCs and, at the same time, redirects HIV from the canonical receptors to the RGD-binding integrins α5β1, αvβ3 and αvβ5. These integrins are highly expressed by DCs and macrophages [65]–[71] and by activated endothelia [17], [22]–[28], which mediate the extravasation of activated lympho-monocyte at inflammation sites [72]. In addition, these effects of Tat are observed in the pico-nanomolar protein range, consistent with the levels detected in sera and tissues of HIV infected subjects [14]–[21], strongly suggesting that they are biologically relevant.

The present data indicate that Tat can increase the chances of acquiring HIV infection by increasing virus infectivity of DCs and other key cells at the portals of entry, and, at the same time, by driving the virus to mucosal sites rich of these cells, which, in turn, activate target T cells present at, or proximal to, the virus portals of entry [73]. This would favor the creation of a local focus of self-perpetrating infection, increasing productive infection and virus dissemination to regional lymph nodes, while limiting the dispersion of viral progeny in tissues poor of target cells. Of note, most of the data shown are by using a csy_22_ Tat, which is transactivation-silent. This was necessary to dissect the entry effect of Tat from its transactivation activity. However, since wt Tat protein is also the most potent transactivator of HIV gene expression and replication, this activity will further amplify virus infection and dissemination promoted by extracellular Tat [15].

The Tat/Env complex is stably formed only with trimeric Env molecules which are present in a little number only on viral particles, whereas monomeric gp120, which is shed in large amounts by both HIV and infected cells, is not well recognized by Tat and, therefore, does not compete with the virus for binding extracellular Tat. In this regard, previous data suggested that cell membrane bound Tat specifically binds the V2 loop of monomeric gp120, promoting HIV-1 infection and spreading [74]. In our system, the V2 loop is not required for complex formation, and its deletion increases the stability of the complex by about ten-fold, suggesting that V2 partially shields the Tat binding site and its removal stabilizes the Tat/Env interactions. This is consistent with the role of V2 in shielding the V3 Env region.

The Tat/Env complex formation is mediated by interactions of Tat with the CCR5-binding regions of Env, including the V3 loop and a region of Env partially overlapping the other Env CCR5-binding site, as suggested by modeling-docking calculations and indicated by the direct binding of Tat to a cyclic V3 loop peptide, and by the lack of enhancement of HIV entry in MDDCs by trimeric ΔV3 Env.

Of importance, Tat binds trimeric Env molecules from different HIV clades (A, B, C).

Finally, the presence of Tat abolishes neutralization of Env entry and infection of DCs by HIV sera lacking anti-Tat Abs. However, when anti-Tat Abs are also present, neutralization is not only restored but further increased. The same is observed with sera from anti-Tat/Env vaccinated monkeys.

The present findings have major implications for both HIV pathogenesis as well as vaccine design: i) the binding of Tat to Env may be pivotal to the establishment of a persistent infection in the host by increasing the local virus load using cells that are key at the portal of entry and by sustaining virus replication and dissemination into tissues [73]; of note, these are the cells that become the virus reservoir in chronic infection; and ii) since Tat binds Env in a region that includes both the CCR5-binding sites, the complex may act by shielding key epitopes for neutralizing Abs induced by the virus during natural infection or elicited by Env-based vaccines. Further, the Tat/Env complex interaction will also likely expose new Env epitopes that are unlikely to be recognized by Abs elicited by Env alone in preventative vaccine approaches. This may explain the lack or marginal protection observed with Env-based vaccines in phase III efficacy studies [1], [2], [3].

Thus, extracellular Tat has a key function to secure HIV infection by redirecting the virus to cells capable of establishing productive infection, HIV dissemination, and virus reservoirs and, at the same time, by promoting virus escape from Env neutralization. Paradoxically, in the absence of anti-Tat Ab responses, as occurring in most HIV infected individuals, these mechanisms may be further increased by anti-Env Abs alone since block of virus entry via DC-SIGN may favor, in the presence of Tat, the integrin-mediated virus entry pathway. By binding surface glycoproteins HIV Tat might contribute to enhance susceptibility to co-infecting pathogens in HIV infected patients. On the other hand, the Tat/Env complex is highly stabilized upon V2 loop deletion, which resembles the reported V1/V2 shortening of early virus isolates in newly infected subjects [75]-[78]. Since V1/V2 deletions are known to expose both the V3 and the other CCR5 binding region of Env to both the co-receptor and to specific neutralizing Abs [44], [79]–[82], these isolates may form the most stable complex with Tat in vivo, be facilitated for transmission and, at the same time, be shielded from the mounting humoral response. This may also explain the correlation of the anti-Tat humoral response with non progression to disease found in both cross-sectional and longitudinal studies in HIV-infected individuals [38], [83]. At the same time, the present data shed light on the mechanisms of protection of monkeys vaccinated with the native Tat protein [4]–[9], [39], the protection from high dose virus challenge of monkeys vaccinated with replication-competent adenoviruses expressing Tat and Env [40], as well as the promising efficacy results seen in the phase II therapeutic trial with Tat in HAART-treated subjects [13]. Of note, anti-Tat Abs from vaccinated and HIV-infected individuals are effective in blocking Tat/Env entry and infection of DCs, indicating that proper anti-Tat Abs can be induced by vaccination. Since anti-Env Abs are already present in HIV-1 infected individuals, these data suggest that Tat alone should be administered for therapeutic vaccine strategies, whereas both Tat and Env should be given combined for preventative vaccination.

In view of these results, a new phase II trial has been initiated in South Africa in HAART-treated patients (ISS T-003, ClinicalTrials.gov NCT01513135), and complexes of Tat and ΔV2 Env are being tested in a multicentric preventative phase I trial in Italy (ISS P-002, ClinicalTrials.gov NCT01441193, http://www.hiv1tat-vaccines.info).

## Supporting Information

Figure S1 Wt Tat, but not cys_22_ Tat, transactivates HIV-1 LTR in TZM-bl cells. RLU in HIV-1 LTR expressing TZM-bl cells cultured in the presence of buffer, or 0.1, 1, 10 µM wt Tat or cys_22_ Tat.
(TIF)

Figure S2 Entry of wt Tat in MDDCs in the presence of different Env molecules and block by anti-integrins antibodies. Clade B trimeric wt Env (twt Env), trimeric ΔV2 Env (tΔV2 Env), monomeric wt Env (mwt Env), or monomeric ΔV2 Env (mΔV2 Env) molecules or buffer were incubated with Tat and added to MDDCs pre-treated with anti-integrin mAbs or a control isotype mAb. Cells were then stained for intracellular Tat and results expressed as the percentage of Tat-positive cells.
(TIF)

Figure S3 Block of trimeric ΔV2 Env or Tat/ΔV2 Env entry in MDDCs by anti-DC-SIGN and anti-integrins antibodies. (**A**) MDDCs from two different donors were pre-incubated with different concentration of an anti-DC-SIGN mAb (20 or 50 µg/mL) or with a control isotype mAb and then 70 nM (donor 1) or 35 nM (donor 2) trimeric ΔV2 Env (SF162) were added for 10 min prior to Env intracellular staining and flow cytometry analysis. (**B**) MDDCs were pre-incubated with an anti-DC-SIGN mAb (50 µg/mL), with anti-integrin mAbs (10 µg/mL each) or a control isotype mAb, and then trimeric ΔV2 Env (70 nM) (SF162) was added for 10 min prior to Env intracellular staining and flow cytometry analysis. (**C**) MDDCs were pre-incubated with anti-DC-SIGN mAb (50 µg/mL), anti-integrin mAbs (10 µg/mL each), both, or a control isotype mAb and then incubated for 10 min with trimeric ΔV2 Env (70 nM) previously complexed with Tat at different concentrations (50, 16.67 and 5.56 nM) prior to Env intracellular staining and flow cytometry analysis. Results are expressed as the percentage of Env-positive cells.
(TIF)

Figure S4 Structural model of the ΔV1-2 Env/Tat binary complex. Color code: Env: dark blue; Env V3-loop: light blue; Tat: yellow; Tat cysteine-rich region: orange; and Tat RGD segment: red. (**A**) panels: surface representation; (**B**) panels: cartoon representation. See experimental procedures for details.
(TIF)

Figure S5 Structural Model of the ΔV1-2 Env/Tat/Integrin αvβ3 Ternary Complex. Color code: ΔV1-2 Env: violet; Tat: yellow; integrin αvβ3: cyan.
(TIF)

Figure S6 Blockade of Tat/Env complex entry into MDDCs by anti-Tat antibodies. Trimeric ΔV2 Env was incubated with PBS, a pool of sera from six HIV uninfected healthy donors, the same pool of sera plus the anti-Tat 2A4.1 mAb, Tat, or Tat plus 2A4.1 mAb, and added to cells. Cells were then stained for intracellular Env and analyzed by flow cytometry. The percentage of Env- positive cells is shown.
(TIF)

Figure S7 Blockade of Tat/Env complex binding in PBL by anti-CD4 antibodies. PBLs were pre-incubated with buffer or an anti-CD4 mAb and then incubated with twt Env or with twt Env which had been pre-incubated with the indicated amounts of Tat. PBLs were then stained with an anti-gp120 mAb and analyzed by flow cytometry. The percentage of Env-binding cells is shown.
(TIF)

Figure S8 Env and Tat interacting residues according to modeling docking analyses. Upper panel: Env interacting residues in the five lowest energy solutions. Residues involved in interactions are indicated by boxes. Different box colors correspond to different solutions. Secondary structure elements are colored as follows: the ΔV1-2 gp120 inner domain of Env substructures are represented in yellow (α1 helix), red (bridging sheet strands), white (three-strand sheet), purple (outer/inner domain transition), and green (α5 helix), while the outer domain is depicted in blue. Lower panel: Tat interacting residues in the five lowest energy solutions. Residues involved in interactions are indicated by boxes in five different colors. Tat regions are colored as follows: purple (N-terminal region), green (cysteine-rich region), yellow (protein core), red (basic region), and grey (C-terminal region).
(TIF)

Table S1 Vaccine protocol design and schedule of immunization of cynomolgus monkeys.
(DOCX)

Table S2 Structures used as templates to model the structures of Tat and Env.
(DOC)

Table S3 Parameters used to perform MD simulations on the V3 loop of the Env protein.
(DOC)

Table S4 Parameters used to perform docking calculations.
(DOC)

Table S5 Parameters used to perform docking calculations.
(DOC)

Table S6 CD4^+^ T cell counts, plasma viral load and proviral DNA load in blood, inguinal lymph nodes and rectal mucosal tissues at 4 week after intrarectal challenge with 70 MID_50_ of SHIV_SF162P4cy_.
