# Supplementary figures and images for: HIV-1 Tat Promotes Integrin-Mediated HIV Transmission to Dendritic Cells by Binding Env Spikes and Competes Neutralization by Anti-HIV Antibodies
Source: PLoS One. 2012 Nov 13;7(11):e48781. doi: 10.1371/journal.pone.0048781 (PMC3496724; doi:10.1371/journal.pone.0048781)

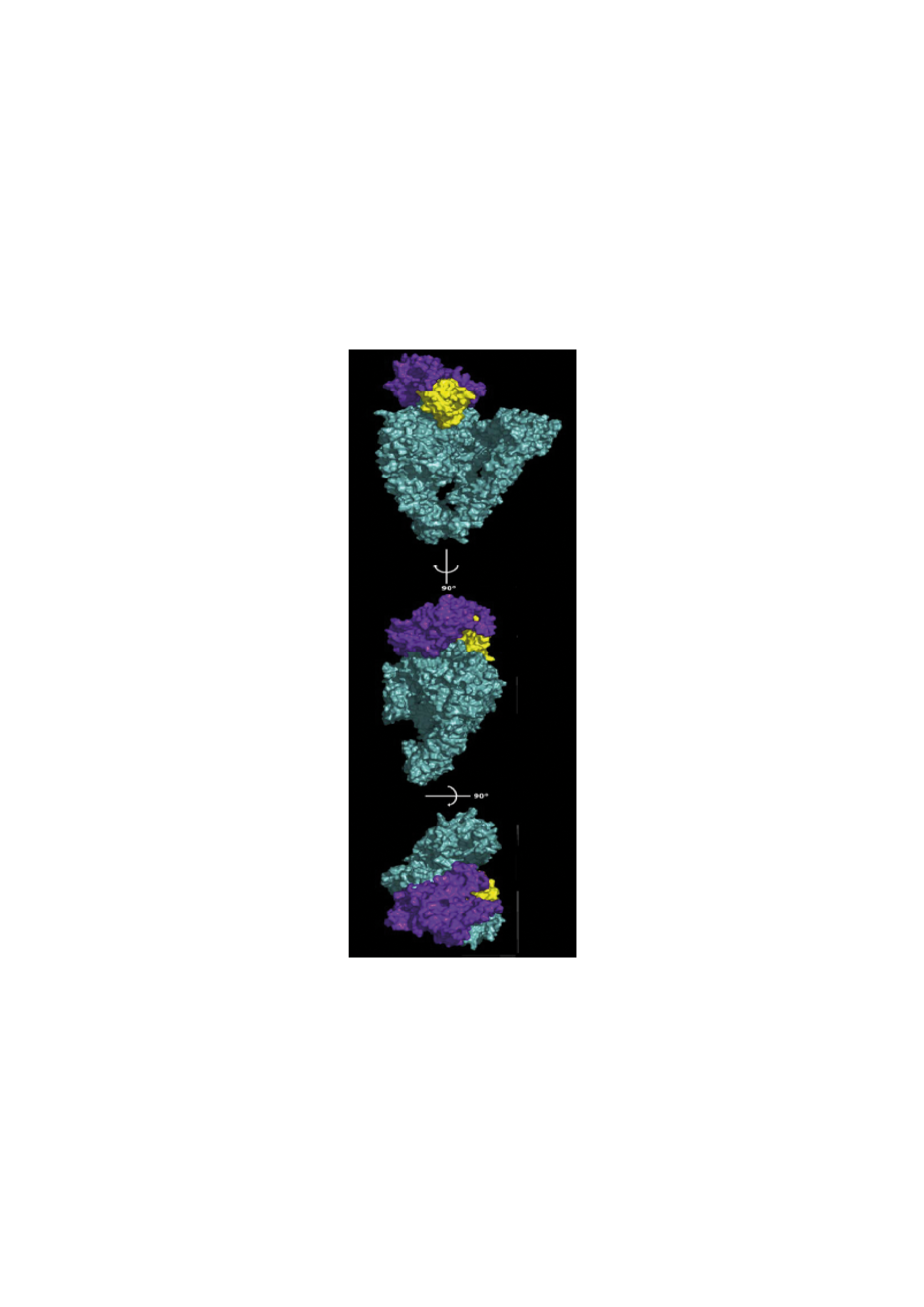

Supplement: Figure S5 — Structural Model of the ΔV1-2 Env/Tat/Integrin αvβ3 Ternary Complex. Color code: ΔV1-2 Env: violet; Tat: yellow; integrin αvβ3: cyan. (TIF) [file pone.0048781.s005.tif]

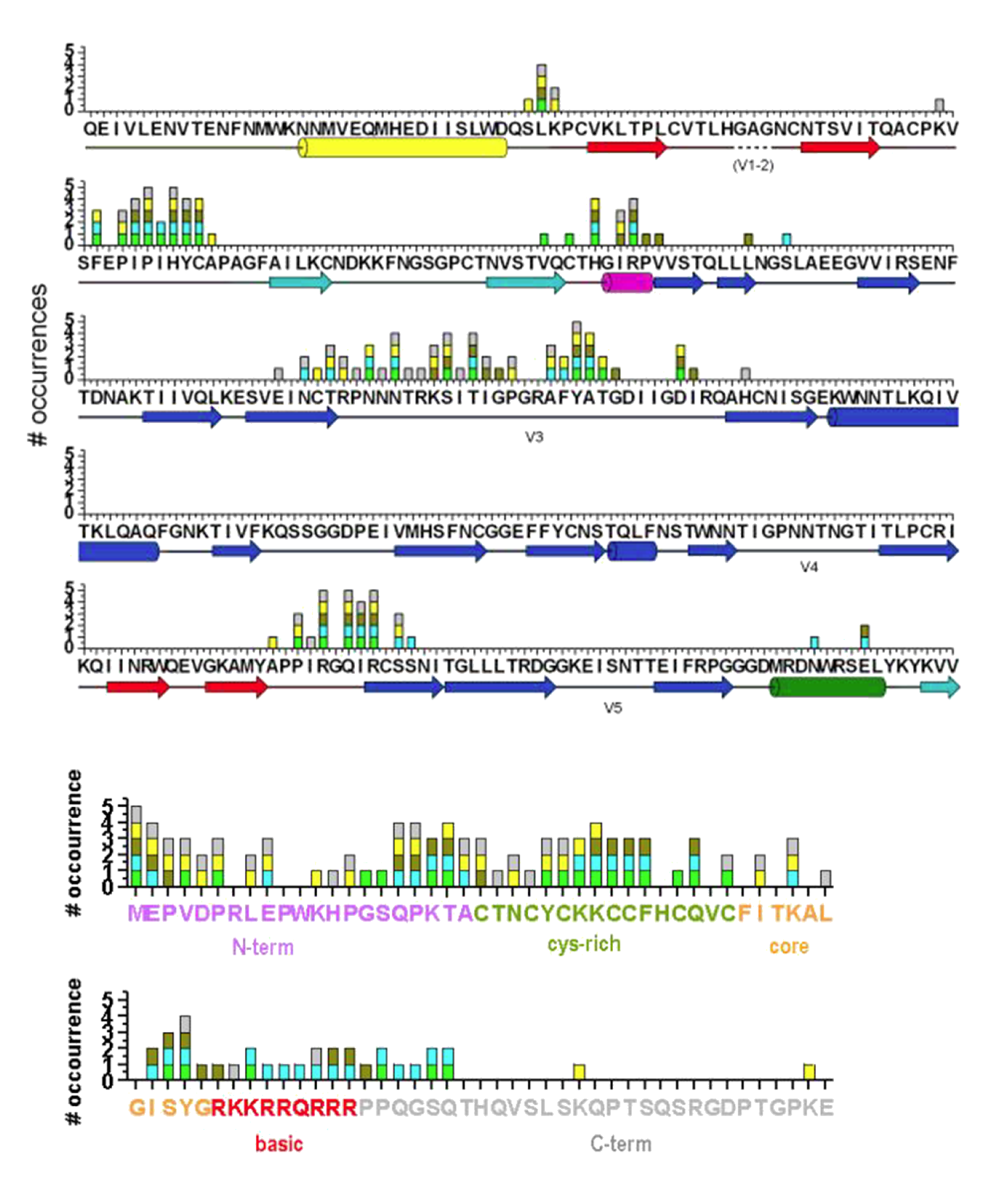

Supplement: Figure S8 — Env and Tat interacting residues according to modeling docking analyses. Upper panel: Env interacting residues in the five lowest energy solutions. Residues involved in interactions are indicated by boxes. Different box colors correspond to different solutions. Secondary structure elements are colored as follows: the ΔV1-2 gp120 inner domain of Env substructures are represented in yellow (α1 helix), red (bridging sheet strands), white (three-strand sheet), purple (outer/inner domain transition), and green (α5 helix), while the outer domain is depicted in blue. Lower panel: Tat interacting residues in the five lowest energy solutions. Residues involved in interactions are indicated by boxes in five different colors. Tat regions are colored as follows: purple (N-terminal region), green (cysteine-rich region), yellow (protein core), red (basic region), and grey (C-terminal region). (TIF) [file pone.0048781.s008.tif]
