# Supplementary material for: HIV-1 Tat Promotes Integrin-Mediated HIV Transmission to Dendritic Cells by Binding Env Spikes and Competes Neutralization by Anti-HIV Antibodies
Source: PLoS One. 2012 Nov 13;7(11):e48781. doi: 10.1371/journal.pone.0048781 (PMC3496724; doi:10.1371/journal.pone.0048781)
Supplement: Table S1 — Vaccine protocol design and schedule of immunization of cynomolgus monkeys. (DOCX) [file pone.0048781.s009.docx]

# Table S1. Vaccine protocol design and schedule of immunization of cynomolgus monkeys.

| **Monkey code** | **Groups** | **Priming (x2)** | **Boost (x1)** | **Boost (x2)** |
| --- | --- | --- | --- | --- |
|  |  | **Tat +ΔV2-Env**  **300 μL, intranasal** | **Tat +ΔV2-Env**  **300 μL, intranasal** | **Tat + ΔV2-Env**  **500 μL, subcute** |
| AH482 | Vaccinated | ΔV2-Env (100 µg)  + Tat (10 µg)  + LT-K63 (30 µg) | ΔV2-Env (100 µg)  +Tat (10 µg)  + LT-K63 (30 µg) | ΔV2-Env (100 µg)  +Tat (10 µg)  + Alum (250 µL) |
| AI011 |  |  |  |  |
| AH651 |  |  |  |  |
| AH979 |  |  |  |  |
| AL128 |  |  |  |  |
| AH590 |  |  |  |  |
| AH694 | Controls | LT-K63 (30 µg) | LT-K63 (30 µg) | Alum (250 µL) |
| AK407 |  |  |  |  |
| AK484 |  |  |  |  |
| AK803 |  |  |  |  |
| AK952 |  |  |  |  |
| AL963 |  |  |  |  |
