# Supplementary material for: HIV-1 Tat Promotes Integrin-Mediated HIV Transmission to Dendritic Cells by Binding Env Spikes and Competes Neutralization by Anti-HIV Antibodies
Source: PLoS One. 2012 Nov 13;7(11):e48781. doi: 10.1371/journal.pone.0048781 (PMC3496724; doi:10.1371/journal.pone.0048781)
Supplement: Table S2 — Structures used as templates to model the structures of Tat and Env. (DOC) [file pone.0048781.s010.doc]

**Table S2.**Structures used as templates to model the structures of Tat and Env

| **Model** | **Template PDB code** | **Region used as Template** |
| --- | --- | --- |
| Tat BH10 | 1jfw | aa 1 - 86 (average structure) |
| Tat BH10 | 1k5k | aa 1 - 87 (average structure) |
| Tat BH10 | 1tbc | aa 1 - 86 (average structure) |
| gp120 domain ΔV1-2 sf162 | 2bf1 | aa 64 – 449 (chain A) |
| gp120 domain ΔV1-2 sf162 | 2b4c | aa 293-334 (chain G) |
