# Supplementary material for: HIV-1 Tat Promotes Integrin-Mediated HIV Transmission to Dendritic Cells by Binding Env Spikes and Competes Neutralization by Anti-HIV Antibodies
Source: PLoS One. 2012 Nov 13;7(11):e48781. doi: 10.1371/journal.pone.0048781 (PMC3496724; doi:10.1371/journal.pone.0048781)
Supplement: Table S3 — Parameters used to perform MD simulations on the V3 loop of the Env protein. (DOC) [file pone.0048781.s011.doc]

**Table S3. Parameters used to perform MD simulations on the V3 loop of the Env protein.**

| Temperature | 300K |
| --- | --- |
| Cut off for the evaluation of the non-bonded interactions | 10 Å |
| Force field | PARM96 |
| Time step | 1.5 fs |
| Water model | TIP3P |
| MD length | 10 ns |

To analyze the V3 loop mobility, extensive molecular dynamics (MD) simulations were performed with the program AMBER 8.0 on the Env protein, where only the V3 loop was allowed to move. The solvent molecules were initially equilibrated by energy minimization and subsequently by performing 15 ps of MD. Temperature was initially increased from 0 to 300 K and then maintained constant for the whole simulation time, coupling the protein to a thermal bath.
