# Supplementary material for: HIV-1 Tat Promotes Integrin-Mediated HIV Transmission to Dendritic Cells by Binding Env Spikes and Competes Neutralization by Anti-HIV Antibodies
Source: PLoS One. 2012 Nov 13;7(11):e48781. doi: 10.1371/journal.pone.0048781 (PMC3496724; doi:10.1371/journal.pone.0048781)
Supplement: Table S4 — Parameters used to perform docking calculations. (DOC) [file pone.0048781.s012.doc]

**Table S4. Parameters used to perform docking calculations**

|  | First calculation | Second calculation | Third calculation |
| --- | --- | --- | --- |
|  | (Tat modelled on 1jfw) | (Tat modelled on 1tbc) | (Tat modelled on 1k5k) |
| Active residues on Env | 15,25,35,36,37,43,64,66,67, 68,69,70,71,73,74,75,76,102, 104,107,110,120,122,157,159,160,161,63,164,165,167,169, 170,171,172,173,175,176,177,178,179,180,181,182,183,273,280,282,284,286,289,290,292,294,296,298,333 | 15,25,35,36,37,43,64,66,67, 68,69,70,71,73,74,75,76,102, 104,107,110,120,122,157,159,160,161,63,164,165,167,169, 170,171,172,173,175,176,177,178,179,180,181,182,183,273,280,282,284,286,289,290,292,294,296,298,333 | 15,25,35,36,37,43,64,66,67, 68,69,70,71,73,74,75,76,102, 104,107,110,120,122,157,159,160,161,63,164,165,167,169, 170,171,172,173,175,176,177,178,179,180,181,182,183,273,280,282,284,286,289,290,292,294,296,298,333 |
| Passive residue on Tat | 1,9,19,20,28,29,32,35,41,46, 49,51,55,56,57,60,61,70,71,85 | 1,3,4,8,12,17,20,21,22,26,29, 31,33,45,46,52,55,58,71,84 | 3,4,7,8,10,18,24,26,29,30,35, 40,49,50,52,53,59,72,73,75,76 |
| Number of complexes calculated in the first stage (randomization of orientations and rigid body energy minimization) | 1000 | 1000 | 1000 |
| Number of complexes calculated in the second stage (semi-flexible simulated annealing in torsion angle space) | 200 | 200 | 200 |
| Number of complexes calculated in the third stage (water refinement) | 200 | 200 | 200 |
| Clustering threshold value (Å) | 2.8 | 2.0 | 2.5 |

The program HADDOCK 1.3 was used to perform ambiguous restraints driven docking calculations for the Tat/Env adduct. Three different calculations were performed using as input the three different models of the Tat protein and 50 snapshots of the MD simulation (one every 200 ps) for the monomeric ΔV1-2 Env, all the residues having solvent accessibility greater than 50%, as calculated by the program NACCESS, and falling at least into one of the interfaces obtained from the initial BIGGER/ClusPro. Calculations were selected as active for Env and as passive for Tat.
