# Supplementary material for: HIV-1 Tat Promotes Integrin-Mediated HIV Transmission to Dendritic Cells by Binding Env Spikes and Competes Neutralization by Anti-HIV Antibodies
Source: PLoS One. 2012 Nov 13;7(11):e48781. doi: 10.1371/journal.pone.0048781 (PMC3496724; doi:10.1371/journal.pone.0048781)
Supplement: Table S5 — Parameters used to perform docking calculations. (DOC) [file pone.0048781.s013.doc]

**Table S5. Parameters used to perform docking calculations**

1. **Docking parameters and ambiguous restraints**

|  | First calculation | Second calculation | Third calculation |
| --- | --- | --- | --- |
|  | (Tat modelled on 1jfw) | (Tat modelled on 1tbc) | (Tat modelled on 1k5k) |
| Ambiguous Active residues on integrin | 150,178,212,213,215,217,1123,1218,4001 | 150,178,212,213,215,217,1123,1218,4001 | 150,178,212,213,215,217,1123,1218,4001 |
| Ambiguous Passive residue on integrin | 146,148,211,214,216,247,248,1125,1126,1179,1180,1312, 1313,1316,4002 | 146,148,211,214,216,247,248,1125,1126,1179,1180,1312, 1313,1316,4002 | 146,148,211,214,216,247,248,1125,1126,1179,1180,1312, 1313,1316,4002 |
| Ambiguous Active residues on Tat | 78,79,80 | 78,79,80 | 78,79,80 |
| Ambiguous Passive residue on Tat | 10,26,28,32,38,41,42,49,50, 51,53,56,57,70,71,73,75,76, 84,85 | 12,17,50,51,52,55,57,58,59, 61,71,72,77,81,84,86 | 10,35,39,40,42,43,49,50,51, 52,53,56,57,71,72,73,74,75, 76,77,82,85 |
| Number of complexes calculated in the first stage (randomization of orientations and rigid body energy minimization) | 1000 | 1000 | 1000 |
| Number of complexes calculated in the second stage (semi-flexible simulated annealing in torsion angle space) | 200 | 200 | 200 |
| Number of complexes calculated in the third stage (water refinement) | 200 | 200 | 200 |
| Clustering threshold value (Å) | 1.5 | 1.5 | 1.5 |

The program HADDOCK 1.3 was used to perform ambiguous restraints driven docking calculations for the Tat-α_v_β_3_ adduct. Three different calculations were performed using as input the three different models of the Tat protein and the integrin structure.

**(B) Unambiguous restraints.**

| Tat residue | Atom type | Integrin residue | Atom type | Distance restrain | Tolerance |
| --- | --- | --- | --- | --- | --- |
| 78 | NH1 | 150 | OD2 | 3.32 Å | ±0.10Å |
| 78 | OD2 | 218 | OD1 | 3.20 Å | ±0.10 Å |
| 78 | NH2 | 218 | OD2 | 2.76 Å | ±0.10 Å |
| 79 | C | 1216 | O | 3.90 Å | ±0.10 Å |
| 80 | OD1 | 4001 | MN2 | 2.64 Å | ±0.10 Å |
| 80 | OD2 | 1122 | N | 2.81 Å | ±0.10 Å |
| 80 | OD2 | 1215 | OD1 | 3.27 Å | ±0.10 Å |
| 80 | N | 1216 | O | 3.46 Å | ±0.10 Å |

The unambiguous restrains are the same for the three calculations.
