# Supplementary material for: HIV-1 Tat Promotes Integrin-Mediated HIV Transmission to Dendritic Cells by Binding Env Spikes and Competes Neutralization by Anti-HIV Antibodies
Source: PLoS One. 2012 Nov 13;7(11):e48781. doi: 10.1371/journal.pone.0048781 (PMC3496724; doi:10.1371/journal.pone.0048781)
Supplement: Table S6 — CD4+ T cell counts, plasma viral load and proviral DNA load in blood, inguinal lymph nodes and rectal mucosal tissues at 4 week after intrarectal challenge with 70 MID50 of SHIVSF162P4cy. [file pone.0048781.s014.doc]

**Table S6. CD4+ T cell counts, plasma viral load and proviral DNA load in blood, inguinal lymph nodes and rectal mucosal tissues at 4 week after intrarectal challenge with 70 MID_50_ of SHIV_SF162P4cy_**

| GROUP | MK | Blood | | |  | Biopsies | | |
| --- | --- | --- | --- | --- | --- | --- | --- | --- |
|  |  | CD4+ cell number*  (/mm^3^) | Plasma viremia  (Eq/mL x 10^-4^) | Proviral DNA  (copies/µg genomic DNA) | |  | Lymph node proviral load  (copies/µg genomic DNA) | Rectum proviral load  (copies/µg genomic DNA) |
| ∆V2 ENV + TAT | AH482 | 1154 (1082) | <50 | 32 | |  | 15 | 90 |
|  | AI011 | 1030 (1994) | <50 | 4 | |  | 3 | 11 |
|  | AH651 | 2005 (2521) | 1.2x10^2^ | 59 | |  | 76 | 129 |
|  | AH979 | 1806 (1379) | <50 | 0 | |  | 0 | 0 |
|  | AL128 | 2424 (1422) | 4.57x10^2^ | 189 | |  | 139 | 96 |
|  | AH590 | 1729 (1027) | <50 | 0 | |  | 3 | 2 |
| CONTROLS | AH694 | 2965 (2141) | 4.26x10^3^ | 118 | |  | 272 | 130 |
|  | AK407 | 1696 (1587) | 5.24x10^2^ | 53 | |  | 516 | 18 |
|  | AK484 | 1661 (1072) | 3x10^2^ | 31 | |  | 23 | 10 |
|  | AK803 | 991 (1314) | 7.9x10^3^ | 48 | |  | 222 | 153 |
|  | AK952 | 2129 (2692) | 3.54x10^3^ | 39 | |  | 253 | 47 |
|  | AL693 | 1288 (1454) | 3.13x10^3^ | 62 | |  | 367 | 173 |

i.r.: intrarectally; * Number in parenthesis indicates the value at the day of challenge
